# Regular Aerobic Exercise Can Effectively Ameliorate the Skeletal Muscle and Mitochondrial Function Impairments Caused by *bves* Deficiency in Zebrafish

**DOI:** 10.3390/ijms27125594

**Published:** 2026-06-20

**Authors:** Wanwan Cai, Wanbang Zhou, Xiushan Wu, Junrong Lei, Haochen Wang, Qiong Wu, Song Zhou, Kang Sun, Xiuyan Li, Zhilong Zhang, Jisheng Zhang, Jingying Ouyang, Yongqing Li, Zhigang Jiang, Xianchu Liu, Wuzhou Yuan, Lan Zheng

**Affiliations:** 1 Hunan Key Laboratory of Physical Fitness and Sports Rehabilitation, College of Physical Education, Hunan Normal University, Changsha 410012, China; 202403022@hunnu.edu.cn (W.C.); zwb@hunnu.edu.cn (W.Z.); 202420153072@hunnu.edu.cn (S.Z.); 202420153107@hunnu.edu.cn (K.S.); 202320152955@hunnu.edu.cn (Z.Z.); 2 Ethnic Traditional Sports Studies, College of Physical Education, Hunan Normal University, Changsha 410012, China; 11238@hunnu.edu.cn (J.L.); 14909@hunnu.edu.cn (J.Z.); 202310150305@hunnu.edu.cn (J.O.); 3 The Laboratary of Heart Development Research, College of Life Science, Hunan Normal University, Changsha 410081, China; xiushanwu2003@aliyun.com (X.W.); 202320142783@hunnu.edu.cn (H.W.); wuqiong@hunnu.edu.cn (Q.W.); 202520142997@hunnu.edu.cn (X.L.); liyongqing2002cn@aliyun.com (Y.L.); 201201140149@hunnu.edu.cn (Z.J.); liuxc645@huas.edu.cn (X.L.); 4 Institute for Scientific Sports and Health Promotion, Hunan University of Arts and Science, Changde 415000, China

**Keywords:** CRISPR-Cas9, *bves*, skeletal muscle atrophy, mitochondrial dysfunction, regular aerobic exercise

## Abstract

The Popeye domain-containing protein 1 (Popdc1), also known as Bves, plays a crucial role in maintaining skeletal muscle homeostasis, with its variants leading to limb–girdle muscular dystrophy type R25. Skeletal muscles of patients with the homozygous missense variant of Bves exhibit impaired membrane trafficking, while skeletal muscle fibers in *bves^S191F^* homozygous mutant zebrafish are significantly reduced and disorganized. However, the mechanism by which the absence of *bves* induces skeletal muscle atrophy remains unclear. In this study, we discovered a novel mechanism whereby *bves* deficiency drives skeletal muscle atrophy by disrupting mitochondrial structure and function. Our findings indicate that *bves* knockout leads to a significant decrease in zebrafish’s ability to swim, atrophy of skeletal muscle tissue, loss of cell membrane localization signals, and abnormalities in mitochondrial structure and function. After an 8-week intervention of regular aerobic exercise, the symptoms of skeletal muscle atrophy in *bves* knockout zebrafish were significantly alleviated, and the expression levels of genes and proteins related to mitochondrial were effectively rescued. These findings establish a connection between *bves* deficiency-induced disruption of mitochondrial structure and function and the onset and progression of skeletal muscle tissue atrophy symptoms, thereby laying a molecular foundation for exercise rehabilitation strategies in atrophic myopathy.

## 1. Introduction

Muscle atrophy is defined by a significant decrease in the mass and diameter of muscle fibers [1,2]. This condition is frequently observed as a consequence of various pathological states and presents a considerable health challenge, particularly affecting the quality of life for elderly individuals and those suffering from chronic diseases [3]. The implications of muscle atrophy extend beyond physical appearance, as they can severely impair mobility, strength, and overall well-being. Research to date has extensively examined the genetic, metabolic, and pharmacological pathways that lead to muscle atrophy. Maintaining skeletal muscle homeostasis is a complex process that requires various factors to work in concert. These factors include mechanical loading, neural input, mitochondrial energy production, immune–inflammatory balance, and signals from the endocrine system. Together, they ensure a balanced relationship between protein synthesis and degradation, which is crucial for muscle health [4]. The gene Popeye domain-containing 1 (*popdc1*, *bves*) encodes a specific protein that is localized to the plasma membrane and binds to cyclic adenosine monophosphate (cAMP) [5]. This protein is particularly abundant in striated muscles, such as those in the heart and skeletal system. In animal models, Bves has been identified as a crucial regulator of both the structural integrity and functional capabilities of these muscle types [6]. Previous findings suggest that Bves acts as a negative regulator of cAMP signaling, which is mediated by Adenylate cyclase 9 (Adcy9). This signaling pathway plays a vital role in maintaining muscle mass and functionality. cAMP is recognized as a key player in regulating the intricate relationship between signaling and metabolic processes, particularly concerning mitochondrial ATP production [7]. Disruptions in normal cAMP signaling can lead to mitochondrial dysfunction, which further exacerbates muscle atrophy [8]. Experiments in mice lacking Bves demonstrate a link between the absence of this protein and negative outcomes in muscle physiology, including decreased muscle mass and suboptimal muscle performance [9]. Mutations that result in the loss of function of the *bves* gene have been linked to limb–girdle muscular dystrophy type R25, a rare genetic disorder that is marked by the progressive weakening of the proximal muscles located in the lower limbs. Additionally, this condition can lead to various complications, including atrioventricular block, which highlights the serious implications of this genetic mutation [10]. In a study involving two Japanese patients diagnosed with autosomal recessive limb–girdle muscular dystrophy type R25, researchers identified a novel recurrent homozygous nonsense variant in the *bves* gene. The muscle-related symptoms of this condition typically emerge in the patients’ youth and can last into adulthood. These symptoms generally originate either in the proximal or distal muscles of the lower limbs and often display an asymmetric pattern of muscle involvement, indicating that one side of the body may be affected more than the other [11,12]. Despite these findings, the precise regulatory mechanisms through which mutations in the *bves* gene lead to the development of limb–girdle muscular dystrophy type R25 are still not well understood, leaving a gap in our comprehension of this genetic disorder.

Skeletal muscle fitness and plasticity are crucial determinants of human health and disease. Mitochondria play a vital role in maintaining skeletal muscle energy homeostasis by undergoing adaptive reprogramming to meet the demands imposed by various physiological and pathophysiological stresses. Dysfunction of skeletal muscle mitochondria has been implicated in the pathogenesis of numerous diseases, including muscular dystrophy, atrophy, type 2 diabetes, and age-related sarcopenia [13]. The mechanisms of mitochondrial quality control (MQC), which are essential for preserving mitochondrial function, encompass mitochondrial biogenesis, dynamics and mitophagy [14,15].

Currently, there is no effective method for the prevention and treatment of skeletal muscle atrophy, making it imperative to explore viable treatment options. It is well established that exercise training can enhance muscle protein synthesis and activate signaling pathways that regulate muscle fiber metabolism and function [16]. Consequently, exercise is often employed as a therapeutic approach to address muscle atrophy. Mitochondria play a crucial role in maintaining skeletal muscle homeostasis and bioenergetic metabolism [17]. Being sensitive to contractile signals, exercise can improve mitochondrial function, promote biosynthesis, and ultimately support both cellular and systemic health [16].

In this study, we successfully constructed a *bves* knockout (*bves* KO) zebrafish strain using CRISPR/Cas9 gene editing technology [18,19]. Our findings indicate that the loss of skeletal muscle and the decline in swimming ability in zebrafish are associated with a reduction in Bves protein levels. By employing a loss-of-function strategy, we demonstrated that the absence of *bves* has detrimental effects on mitochondrial structure and function, skeletal muscle mass, and swimming ability performance in zebrafish. Specifically, the lack of *bves* leads to both structural and functional impairments in the skeletal muscle mitochondria, resulting in an imbalance in the expression of complex proteins within the mitochondrial respiratory electron transport chain, which ultimately decreases muscle mass and swimming ability. This is crucial for elucidating the potential mechanisms by which *bves* variants or knockdown contribute to skeletal muscle atrophy. Furthermore, the adult zebrafish, characterized by its well-developed skeletal muscle system and highly controllable motor behavior, presents unique advantages for studying the effects of *bves* deficiency on muscle atrophy and exercise interventions. The robust sustained swimming capacity of adult zebrafish, which can withstand flow velocities tolerance of up to 30 cm/s, allows for precise quantification of exercise intensity (such as velocity–duration gradients) using a customized counter-current device, effectively simulating human progressive resistance training [20]. This facilitates a systematic evaluation of the intervention effects of regular aerobic exercise on myofibrillar remodeling in *bves* KO zebrafish. The results demonstrate that an 8-week regimen of regular aerobic exercise intervention effectively alleviates the symptoms of skeletal muscle atrophy and mitochondrial structural and functional dysfunction caused by *bves* KO in zebrafish. This research has, for the first time, elucidated the mechanism by which Bves maintains skeletal muscle homeostasis through the regulation of mitochondrial structure and function, as well as the effects of regular aerobic exercise on skeletal muscle atrophy. These findings provide an early warning for clinical targeted treatment of skeletal muscle atrophy-related diseases and offer a theoretical foundation for exercise rehabilitation strategies of patients with LGMDR25.

## 2. Results

### 2.1. High Expression of bves in Zebrafish Skeletal Muscle

**Figure 1:**
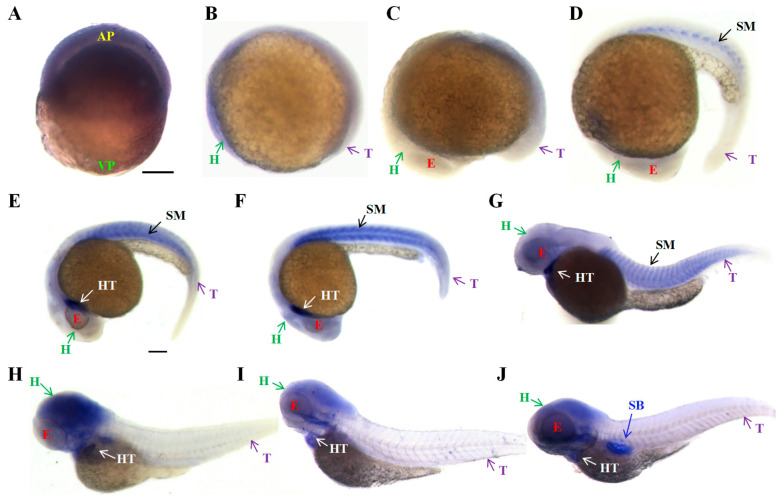
Illustrates the spatiotemporal expression pattern of *bves* in zebrafish embryos as observed through WISH. (**A**) At the 40% epiboly stage, *bves* exhibits ubiquitous expression. (**B**) At the 90% epiboly stage, expression remains ubiquitous. (**C**) By 10 ss, expression is low in the head but high in the dorsal and tail regions. (**D**) At 21 ss, *bves* expression is noted in the head, cardiac primordium, and skeletal muscle. (**E**) At 26 ss, high expression persists in the head, heart, and skeletal muscle. (**F**) At 24 h post-fertilization (hpf), *bves* shows high expression in the head, heart, and skeletal muscle. (**G**) At 48 hpf, expression remains high in the head, heart, and skeletal muscle. (**H**) By 3 dpf, high expression is observed in the head and heart, with downregulated expression in the tail skeletal muscle. (**I**) At 4 dpf, high expression continues in the head and heart, with further downregulation in the tail skeletal muscle. (**J**) By 5 dpf, *bves* is highly expressed in the head, heart, and swim bladder, while expression in the tail skeletal muscle remains downregulated. Abbreviations used are as follows: AP: animal pole. VP: vegetal pole; H: head; T: tail; E: eyes; SM: skeletal muscle; HT: heart; SB: swim bladder. Scale bar = 250 μm.

As previously mentioned, we cloned the open reading frame (ORF) of *bves* and synthesized a DIG-labeled antisense RNA probe for *bves*. The expression pattern of zebrafish *bves* during embryogenesis was analyzed through WISH of embryos. As illustrated in Figure 1, *bves* is ubiquitously expressed during the early stages of embryonic development (Figure 1A–C). Beginning at the 10-somite stage (ss) post-fertilization, it is highly expressed in regions such as the head, heart, and skeletal muscle (Figure 1D–G). From 3 days post-fertilization (dpf), the expression in the skeletal muscle region gradually decreases, while the expression becomes predominantly concentrated in the head and heart regions (Figure 1H,I). At 5 dpf, the *bves* gene is detected not only in the head and heart but also specifically expressed in the swim bladder (Figure 1J). The high expression of *bves* in skeletal muscle tissue during the early stages suggests that it may play a significant role in skeletal muscle development.

### 2.2. Construction of bves KO Zebrafish Line

**Figure 2:**
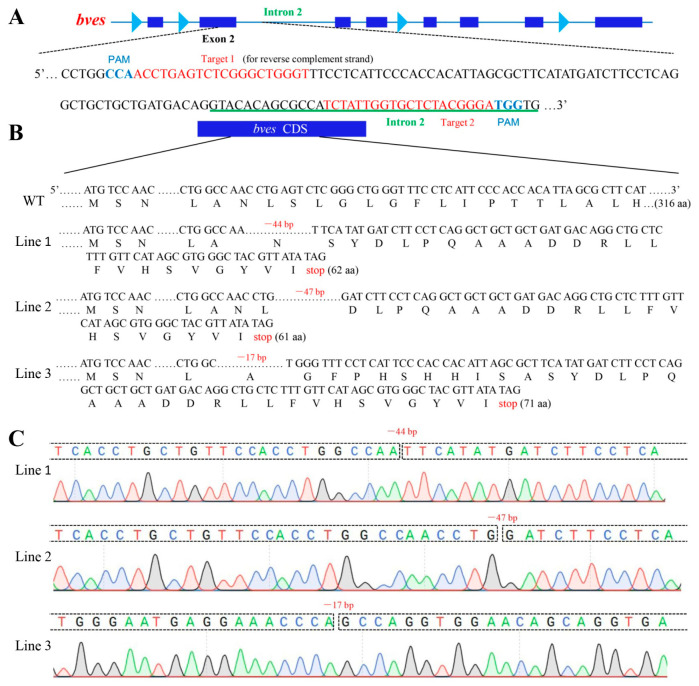
Illustrates the schematic diagrams related to the knockout of the *bves* gene in zebrafish. (**A**) The diagram depicts the sgRNA targeting strategy for the *bves* gene knockout. The blue horizontal line signifies the genomic DNA of *bves*, while the blue rectangles represent the exons of the *bves* gene. The target site sequence is indicated in red font, and the protospacer adjacent motif (PAM) is denoted in blue font. (**B**) This diagram outlines the three heritable mutant alleles of *bves* generated through gene knockout, along with their corresponding encoded protein sequences. (**C**) The sequencing peak diagram for the three *bves* mutant alleles is presented, where the bases A, G, C, and T are represented by green, black, blue, and red curves, respectively.

To investigate the role of the *bves* gene in early zebrafish development, we developed a *bves* KO zebrafish strain. A pair of small guide RNAs (sgRNAs), referred to as Target 1 and Target 2 (Figure 2A), were designed to target exon 2 of *bves*, resulting in three zebrafish lines (Lines 1–3) that exhibit frameshift mutant alleles. Sanger sequencing confirmed the presence of specific mutations: Line 1 had a 44 bp deletion; Line 2 had a 47 bp deletion; and Line 3 had a 17 bp deletion (Figure 2B,C).

### 2.3. bves Deficiency Impairs Zebrafish’s Swimming Ability

**Figure 3:**
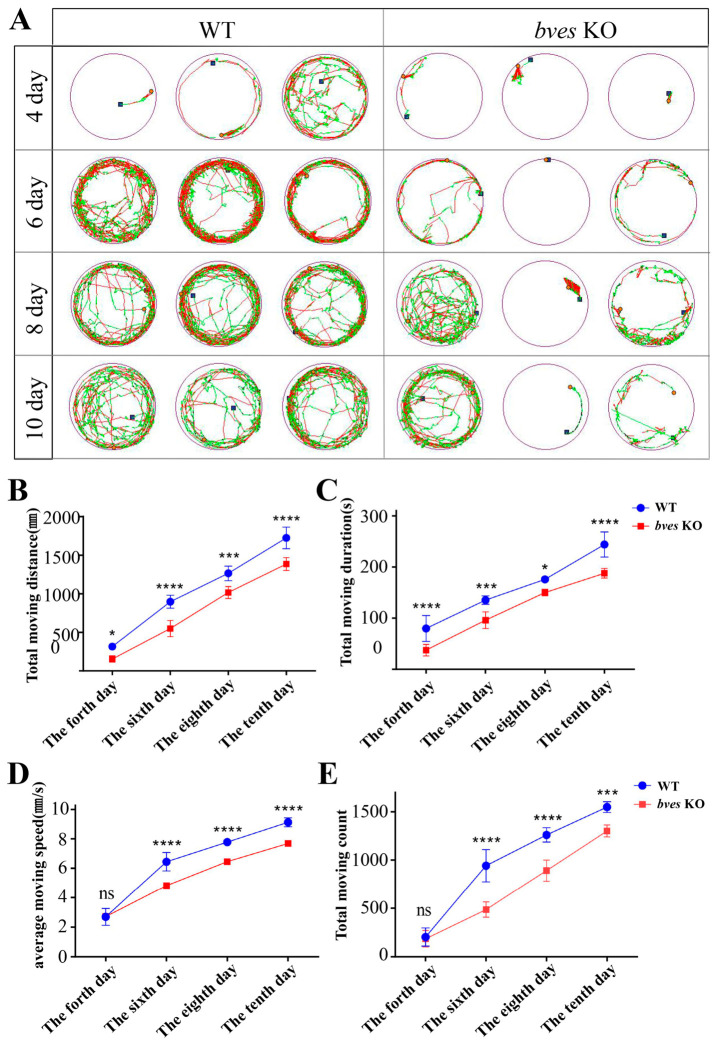
Illustrates the behavioral trajectory of early zebrafish with *bves* gene knockout. The mobile trajectory was significantly reduced in the *bves* KO zebrafish. (**A**) The movement trajectories of zebrafish in different groups. The blue square indicates the starting point, while the orange dot represents the ending point. The red and green lines denote different speeds of the trajectories; the green line indicates medium relative speed, whereas the red line indicates a higher relative speed. (**B**) The total movement distance of zebrafish in different groups. (**C**) The total movement duration of zebrafish in different groups. (**D**) The average movement speed of zebrafish in different groups. (**E**) The total movement count of zebrafish in different groups. The blue line represents the WT group, and the red line represents the *bves* KO group. Data are presented as mean ± SEM. Statistical significance is indicated as * *p* < 0.05, *** *p* < 0.001 and **** *p* < 0.0001. A t-test was employed for statistical analysis.

The motor behaviors and behavioral trajectories of zebrafish embryos were recorded on the 4th, 6th, 8th, and 10th days for a duration of 5 min, with each experiment repeated three times. We captured and documented the activity tracks of each group of zebrafish in a 24-hole culture plate, selecting three holes from each group for display, as illustrated in Figure 3A. The behavioral trajectories and activity durations of zebrafish larvae from the two groups within the culture plate were collected. The total movement distance, total duration, movement speed, and frequency of movement were measured zebrafish in each group.

The behavior trajectories of zebrafish larvae were observed over a period of four days. According to the collected data, both the total moving distance and the frequency of movement in wild-type (WT) and *bves* KO zebrafish exhibited a marked increase with the development of the larvae. Behavioral data from the 4th, 6th, 8th and 10th days were analyzed, revealing that the total movement distance of *bves* KO zebrafish was significantly smaller than that of WT zebrafish, with statistically significant differences (Figure 3B, *n* = 12, *p* < 0.05). Additionally, a statistical analysis of the total movement time indicated that the duration of movement in *bves* KO zebrafish was reduced compared to WT zebrafish (Figure 3C). In the comparison of exercise speed, no significant difference was observed between the two groups on the fourth day (Figure 3D). However, over time, the exercise speed of *bves* KO zebrafish significantly decreased, showing a highly significant difference compared to WT zebrafish (Figure 3D, *p* < 0.0001). The number of movements across the different groups was assessed, and it was found that *bves* KO zebrafish exhibited a significant reduction in the number of movements compared to WT zebrafish over time (Figure 3E).

**Figure 4:**
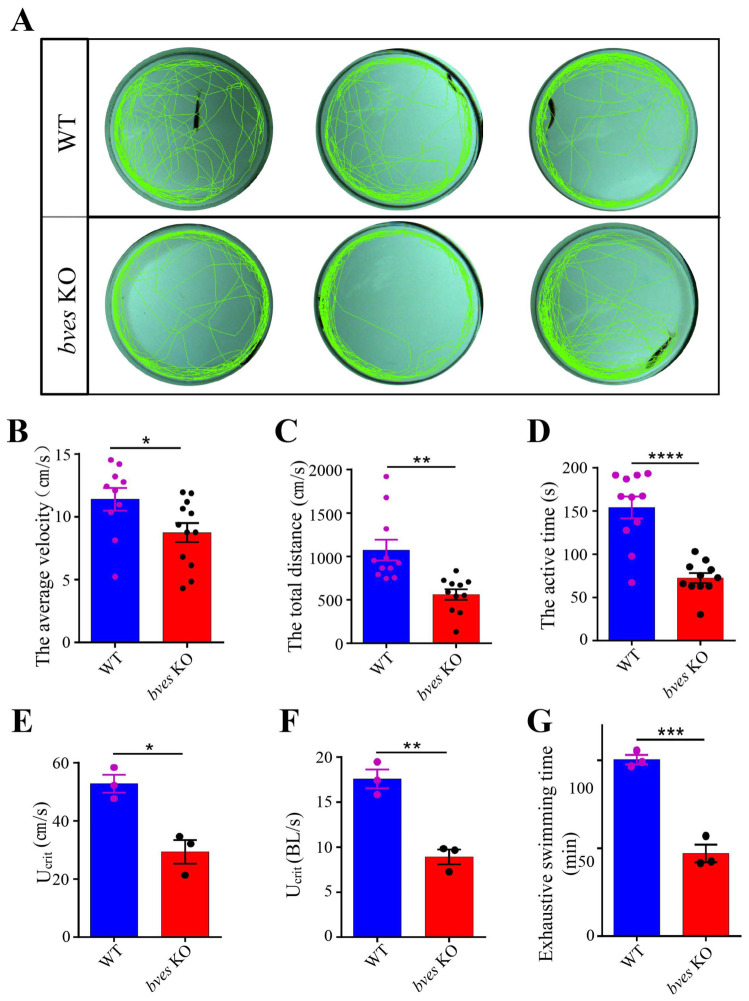
Illustrates the analysis of the movement trajectory and capabilities of adult zebrafish. (**A**) Movement trajectories were recorded (*n* = 11). (**B**) The average velocity of movement was calculated. (**C**) The total distance traveled was measured. (**D**) The active duration of movement was determined. (**E**–**G**) Ucrit, Ucrit-r, and exhaustive swimming time were evaluated using the Loligo System (*n* = 3). Data are presented as mean ± SEM. Statistical significance is indicated as follows: * *p* < 0.05, ** *p* < 0.01, *** *p* < 0.001 and **** *p* < 0.0001. A *t*-test was employed for statistical analysis.

Subsequently, we examined the impact of *bves* loss on adult zebrafish swimming ability. The findings revealed that *bves* KO zebrafish exhibited impaired behavior, characterized by reluctance to move and fewer movement trajectories (Figure 4A). Notably, there were significant decreases in average velocity, total distance traveled, and active time (Figure 4B–D). Additionally, the critical swimming speed (Ucrit), relative swimming speed (Ucrit-r), and exhaustive swimming time during the test phase were significantly reduced in *bves* KO zebrafish (Figure 4E–G). These indicators are commonly utilized to indirectly assess the swimming ability of fish and their performance under increased swimming speeds. In conclusion, the results suggest that *bves* deficiency leads to poor behavioral performance and impairs the swimming ability of zebrafish.

### 2.4. Regular Aerobic Exercise Alleviates Skeletal Muscle Atrophy in bves KO Zebrafish

**Figure 5:**
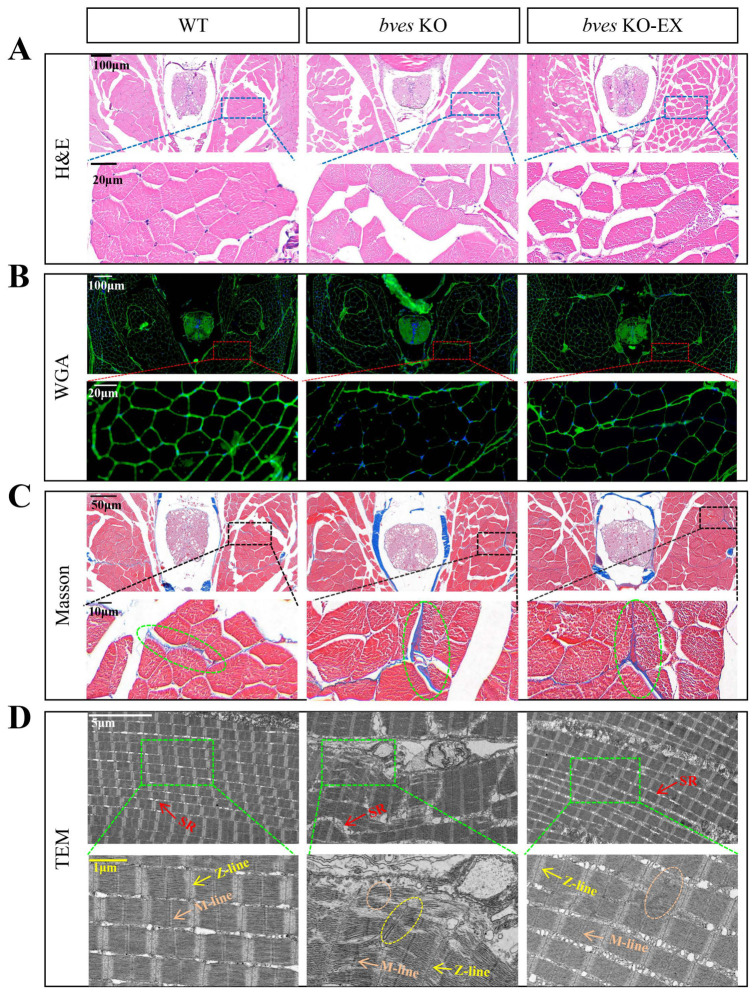
Regular aerobic exercise mitigates skeletal muscle atrophy in *bves* KO zebrafish. (**A**) Displayed are representative micrographs of H&E stained sections, with a scale bar of 100 μm, along with an enlarged view at a scale bar of 20 μm. (**B**) Representative micrographs of WGA stained sections are shown, with a scale bar of 100 μm, and an enlarged view at a scale bar of 20 μm. (**C**) Masson staining was performed to assess collagen deposition, featuring a scale bar of 50 μm and an enlarged view at a scale bar of 10 μm (*n* = 3). (**D**) TEM was utilized to examine the ultrastructure of skeletal muscle, with a scale bar of 5 μm and an enlarged view at a scale bar of 1 μm; SR indicates the sarcoplasmic reticulum.

Subsequently, we investigated the effects of *bves* deficiency on the structure and mass of skeletal muscle in zebrafish. The hematoxylin and eosin (H&E) and wheat germ agglutinin (WGA) staining revealed a decrease in myofiber size (Figure 5A,B and Figure S1). Furthermore, WGA staining indicated a significant reduction in the fluorescent signal of the skeletal muscle cell membrane (Figure 5B), suggesting that *bves* deficiency disrupts membrane localization signals, potentially leading to an abnormal cell membrane structure. Masson staining results demonstrated an increase in collagen deposition in the skeletal muscle of *bves* KO zebrafish (Figure 5C, oval section). Transmission Electron Microscope (TEM) analysis revealed disorganized skeletal muscle fibers in *bves* KO zebrafish, characterized by irregular intermyofibrillar spacing, indistinct light and dark bands of myofibrils, degradation of the M-line, and fractures in the Z-line (Figure 5D, oval section). Next, we aim to further investigate the ameliorative effects of regular aerobic exercise intervention on skeletal muscle atrophy symptoms. The exercise protocol involves placing fish in a custom-designed exercise apparatus, forcing them to swim continuously against a current within a confined area. Each exercise session lasts 1.5 h, performed for 3 consecutive days followed by a rest day. Notably, the phenotypic abnormalities of the mutants significantly improved after eight weeks of regular aerobic exercise intervention. Statistical analysis indicated that the average Feret diameter of skeletal muscle fibers in the WT group was approximately 35 μm. In contrast, the Feret diameter of skeletal muscle fibers in the *bves* KO group was significantly decreased, indicating that the absence of *bves* resulted in a tendency for skeletal muscle fiber atrophy. Conversely, the Feret diameter of skeletal muscle fibers in the *bves* KO-exercised (*bves* KO-EX) group returned to normal levels (Figure S1A). Statistical analysis of the cross-sectional area of individual skeletal muscle fibers revealed that WT zebrafish myofibrils were predominantly found in the range of 500–600 μm^2^. In contrast, *bves* KO zebrafish displayed the highest proportion of myofibrils within the 200–300 μm^2^ range, indicating that *bves* deficiency leads to an atrophic tendency in skeletal muscle cells. Notably, *bves* KO-EX zebrafish exhibited the greatest distribution of myofibrils in the 400–500 μm^2^ range (Figure S1B). These findings suggest that regular aerobic exercise can effectively mitigate the skeletal muscle atrophy associated with *bves* knockout.

### 2.5. RNA-Seq Analysis of the Mechanism of bves Deficiency-Induced Zebrafish Muscle Atrophy

**Figure 6:**
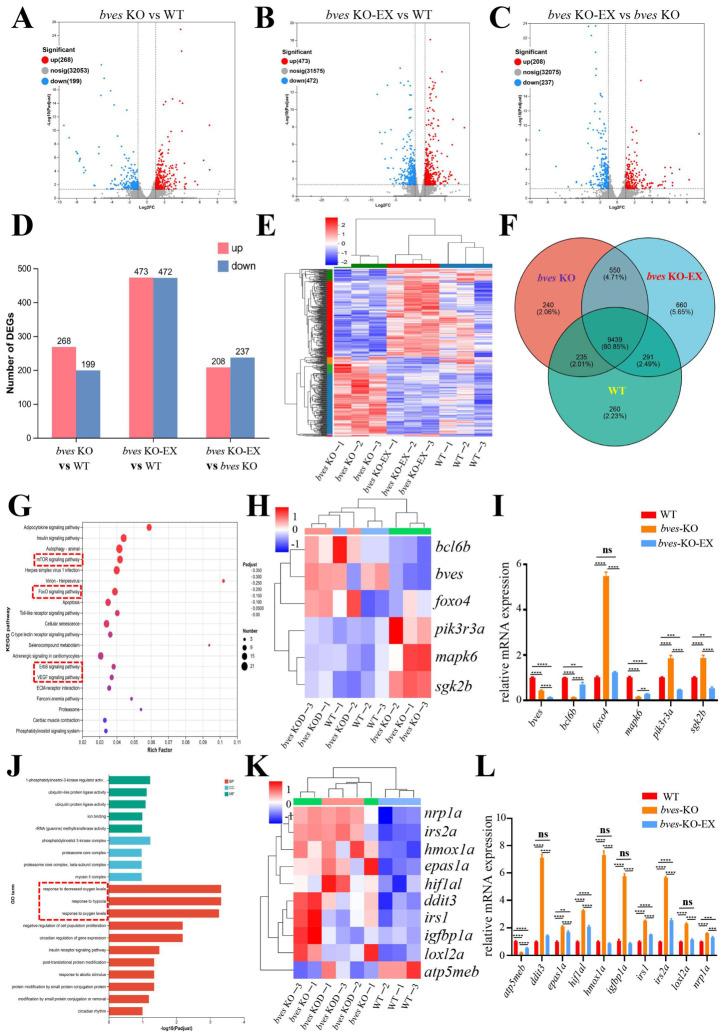
RNA-Seq analysis of the mechanisms underlying muscle atrophy in *bves* KO zebrafish. (**A**–**C**) The volcano plot displays DEGs, where blue dots indicate downregulated genes, red dots signify upregulated genes, and gray dots represent genes exhibiting no significant expression differences across various conditions (significance threshold: |log2FC| > 2.0 and *p* < 0.05). (**D**) Statistical results for DEGs are presented. (**E**) A heatmap of DEGs is shown, with rows representing genes and columns representing samples. The red and blue colors denote high and low expression levels, respectively, while darker colors indicate more pronounced significant differences. (**F**) A Venn diagram depicts the total number of identified genes. (**G**) KEGG pathway enrichment analysis highlights the top 20 pathways, where dot size corresponds to the number of enriched genes and color intensity reflects the level of significance. Red boxes indicate significantly altered signaling pathways related to mitochondrial function. (**H**) The DEGs clustering heatmap illustrates signaling pathways associated with mitochondrial function. (**I**) Quantitative real-time reverse transcription polymerase chain reaction (RT-qPCR) validation confirms the mRNA expression levels of mitochondrial related DEGs. (**J**) GO functional enrichment analysis presents the top 20 categories, including Biological Process (BP), Cellular Component (CC), and Molecular Function (MF). Red boxes highlight entries associated with oxygen concentration (e.g., ‘response to decreased oxygen levels’, ‘response to hypoxia’). (**K**) The DEGs clustering heatmap for oxygen concentration terms are displayed. (**L**) RT-qPCR validation further confirms the mRNA expression levels of oxygen concentration related DEGs (*n* = 6). Statistical significance was determined using one-way analysis of variance (ANOVA), followed by Tukey’s test for pairwise comparisons. Data are presented as mean ± standard error of the mean (SEM). ** *p* < 0.01, *** *p* < 0.001, **** *p* < 0.0001, ns indicates no significant difference.

To fully understand the altered gene expression profiles and the underlying mechanisms in *bves* KO zebrafish skeletal muscle, the skeletal muscle transcriptomes of WT, *bves* KO and *bves* KO-EX zebrafish were analyzed using RNA sequencing. Relative to the WT group, the *bves* KO group exhibited 268 upregulated and 199 downregulated DEGs. The *bves* KO-EX group demonstrated 473 upregulated and 472 downregulated DEGs. When comparing the *bves* KO group to the *bves* KO-EX group, we found 208 upregulated and 237 downregulated DEGs (Figure 6A–D, Padj < 0.05, fold change > 2). All DEGs are displayed in the heatmaps in Figure 6E. The results indicated that 790 genes were specifically expressed, while 551 genes were not detected in the *bves* KO zebrafish skeletal muscle compared to WT zebrafish. The *bves* KO-EX zebrafish skeletal muscle exhibited 660 gene specific expressions when compared to both WT and *bves* KO zebrafish (Figure 6F). KEGG enrichment analyses of the differentially expressed genes revealed significant alterations in mitochondria related pathways within the *bves* KO zebrafish skeletal muscle. Notably, the *bves* KO-EX group demonstrated significant rescue effects (Figure 6G–I). GO enrichment analysis indicated that items related to oxygen concentration ranked the highest. In the *bves* KO group, genes associated with regulating oxygen concentration were dysregulated, but these were effectively rescued in the *bves* KO-EX group (Figure 6J–L). These findings suggest that skeletal muscle atrophy resulting from *bves* deficiency may be closely linked to mitochondrial structure and respiratory function.

### 2.6. Regular Aerobic Exercise Can Alleviate Reactive Oxygen Species (ROS) and Mitochondrial Structural Abnormalities Caused by bves Deficiency

**Figure 7:**
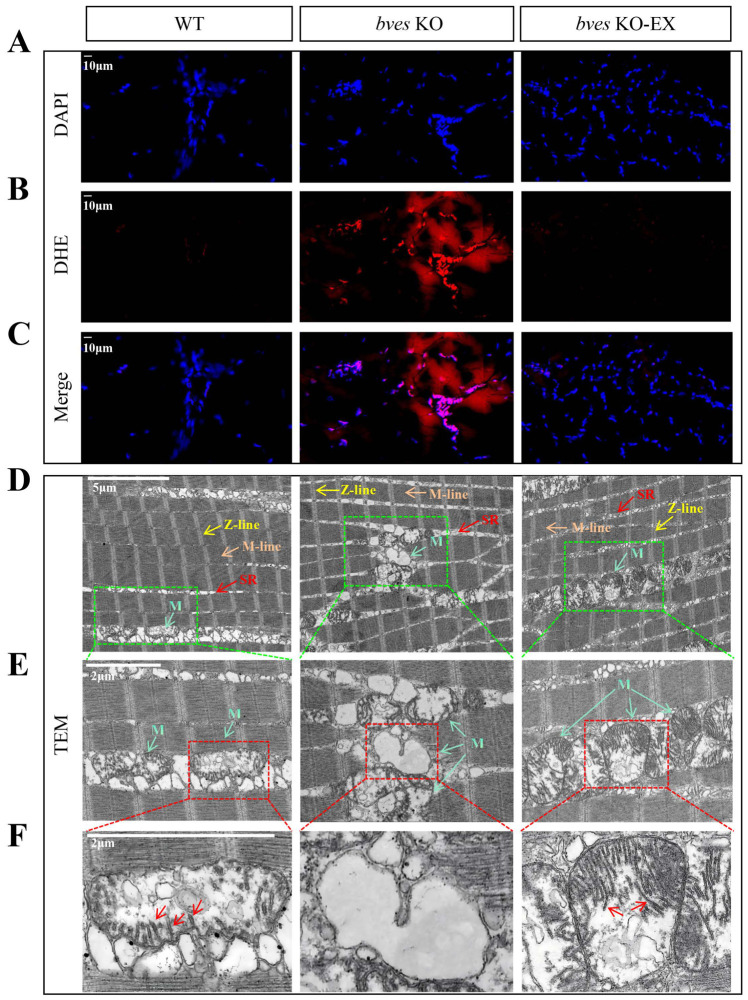
Histological results of mitochondrial structure in skeletal muscle tissue. (**A**) DAPI staining reveals the skeletal muscle tissue, with a scale bar of 10 μm. (**B**) DHE staining also depicts the skeletal muscle tissue, with a scale bar of 10 μm. (**C**) The merged image of DPAI and DHE staining illustrates the skeletal muscle tissue, with a scale bar of 10 μm. (**D**–**F**) TEM observations of zebrafish skeletal muscle mitochondrial ultrastructure are shown, with scale bars of 5 μm and 2 μm; SR indicates the sarcoplasmic reticulum; M represents mitochondria; the red arrow in (**F**) indicates the mitochondrial cristae.

Skeletal muscle atrophy involves significant remodeling of fibers and is characterized by deficits in mitochondrial content and function. An increase in ROS is a hallmark indicator of mitochondrial dysfunction. The dihydroethidium (DHE) staining results indicated that ROS levels in the skeletal muscle of *bves* KO zebrafish were significantly higher than those in the WT group. In contrast, eight weeks of regular aerobic exercise significantly reduced superoxide (O^2−^) levels in the skeletal muscle tissue (Figure 7A–C and Figure S2). Subsequently, it was assessed whether *bves* KO impairs mitochondrial structure and biogenesis. The results revealed that genes involved in the regulation of mitochondrial structure and function were downregulated (Figure S3A). The TEM analysis showed that the skeletal muscle mitochondria in WT zebrafish were either short rod-shaped or spherical, characterized by a high quantity of mitochondria, well-organized and abundant cristae, and a dense mitochondrial matrix. In contrast, the skeletal muscle mitochondria in *bves* KO zebrafish exhibited severe abnormalities, including swelling; membrane rupture; loss of cristae; matrix lysis; reduced quantity; and increased mitochondrial Feret diameter, perimeter and area (Figure 7D–F and Figure S3B–F). These results suggest that the absence of *bves* impairs membrane localization signals and affects the integrity of mitochondrial membrane structure.

### 2.7. Regular Aerobic Exercise Can Alleviate Mitochondrial Respiratory Dysfunction Caused by bves Deficiency

**Figure 8:**
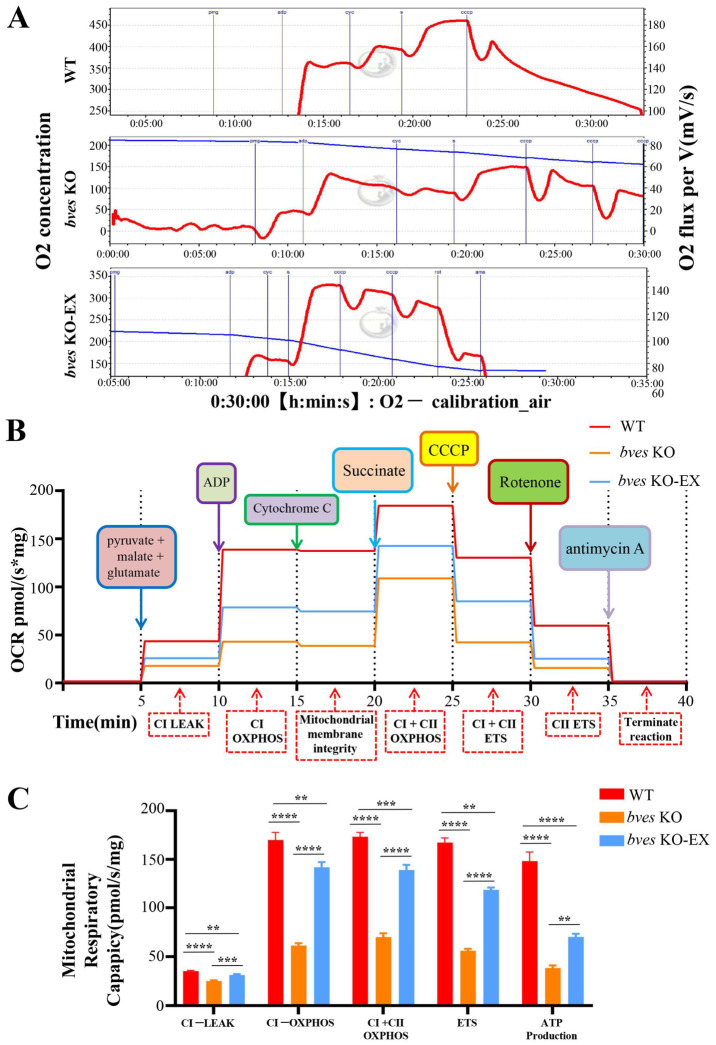
Measurement results of mitochondrial respiratory function in zebrafish skeletal muscle. (**A**) The real-time profile of the oxygen consumption rate (OCR) illustrates mitochondrial respiration. (**B**) The dynamic curve of OCR recorded by the O2K high-resolution respirometer is shown. (**C**) A quantification of mitochondrial respiratory parameters is provided, including complex I leak state (CI leak), complex I oxidative phosphorylation (CI OXPHOS), complex I + II combined oxidative phosphorylation (CI + II OXPHOS), maximal respiratory capacity (ETS), and ATP production capacity (ATP production; *n* = 6). Statistical significance was determined using one-way analysis of variance, followed by Tukey’s test for pairwise comparisons. Data are presented as mean ± standard error of the mean. ** *p* < 0.01, *** *p* < 0.001, **** *p* < 0.0001.

We utilized an O2K cell energy metabolism detector to quantitatively assess mitochondrial respiratory function. Datlab7 software (https://www.oroboros.at/index.php/datlab-7-4-download/, accessed on 2 September 2025) was employed for real-time tracking of mitochondrial respiration and oxygen consumption rates (Figure 8A). Further analysis of mitochondrial respiratory chain function indicated that *bves* knockout resulted in a systematic impairment of respiratory chain function (Figure 8B). Statistical results demonstrated that, compared to the WT group, the *bves* KO group exhibited a significant decrease in the activity of mitochondrial complex I, oxidative phosphorylation levels of mitochondrial complex I and complex I+II, maximum electron transfer chain (ETC) capacity, and ATP production levels. Notably, it is noteworthy that the mitochondrial respiratory function in the *bves* KO-EX group was partially restored (Figure 8C).

**Figure 9:**
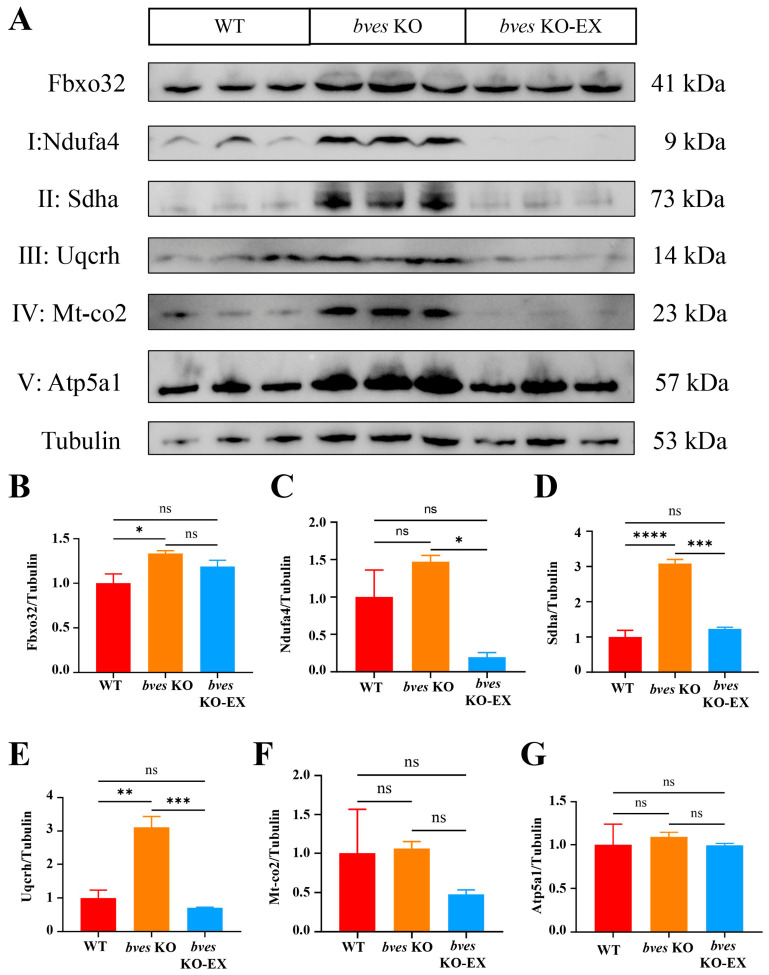
The results of Western blot analysis conducted to assess the expression levels of mitochondrial complex proteins (*n* = 3). (**A**) The Western blot illustrates the protein expressions of Fbxo32, Ndufa4, Sdha, Uqcrh, Mt-co2 and Atp5a1 (*n* = 3). (**B**) The relative expression level of Fbxo32 is shown. (**C**) The relative expression level of Ndufa4 is depicted. (**D**) The relative expression level of Sdha is presented. (**E**) The relative expression level of Uqcrh is illustrated. (**F**) The relative expression level of Mt-co2 is demonstrated. (**G**) The relative expression level of Atp5a1 is displayed. Statistical significance was determined using one-way analysis of variance, followed by Tukey’s test for pairwise comparisons. Data are presented as mean ± standard error of the mean. * *p* < 0.05, ** *p* < 0.01, *** *p* < 0.001, **** *p* < 0.0001, ns indicates no significant difference.

Further Western blot analysis revealed that *bves* gene deletion significantly upregulated the expression of the skeletal muscle atrophy protein Fbxo32 in zebrafish. However, after 8 weeks of regular aerobic exercise, the Fbxo32 protein levels in the *bves* KO-EX group were nearly restored to normal (Figure 9A,B). Subsequently, we assessed the expression levels of five complexes in the mitochondrial respiratory chain. The results demonstrated that in the *bves* KO group, the expression of mitochondrial complex I (Ndufa4), mitochondrial complex II (Sdha), mitochondrial complex III (Uqcrh), mitochondrial complex IV (Mt-co2), and mitochondrial complex V (Atp5a1) all showed an upregulation trend (Figure 9A,C–G). The raw results of all proteins are in Supplementary File S1—Original Protein Results. We speculate that the absence of *bves* leads to mitochondrial membrane swelling and rupture and impaired mitochondrial oxidative respiration function, and it may activate intracellular metabolic compensation mechanisms, resulting in an upregulation trend of electron transport chain complex proteins on the mitochondrial inner membrane. In summary, these results indicate that *bves* deficiency causes an imbalance in the expression of oxidative respiration-related proteins. However, it is noteworthy that the mitochondrial complex levels in most of the *bves* KO-EX group were effectively restored.

The results indicated that aerobic exercise reversed skeletal muscle atrophy induced by *bves* deficiency by restoring protein expression levels of mitochondria-related genes, maintaining mitochondrial structural integrity and rehabilitating the function of the oxidative phosphorylation (OXPHOS) pathway. This finding confirms that the deletion of *bves* results in impaired membrane localization signals and a significant loss of mitochondrial cristae, thereby driving the molecular mechanism of muscle atrophy through the disruption of mitochondrial respiratory function.

## 3. Discussion

Although previous studies have indicated that pathogenic variants of the *bves* gene are associated with LGMDR25, the underlying pathological mechanisms remain unclear. In this study, we elucidate the role of *bves* in regulating mitochondrial structural function and the architecture of skeletal muscle fibers in the context of skeletal muscle atrophy. Our findings demonstrate that *bves* is essential for preserving both the morphology and functionality of skeletal muscle fibers. 

The absence of *bves* resulted in the loss of membrane localization signals in zebrafish skeletal muscle cells, which was accompanied by a significant decrease in skeletal muscle mass and swimming capacity. Importantly, our data demonstrate that *bves* KO zebrafish exhibit impaired mitochondrial morphology and function, as evidenced by extensive loss of mitochondrial cristae membranes, compromised mitochondrial respiratory function, and reduced expression of numerous mitochondria-related genes. This impairment may significantly contribute to skeletal muscle atrophy. Therefore, these findings reveal the crucial role of Bves in protecting mitochondrial function, and maintaining skeletal muscle mass and strength, as well as the pathological mechanisms by which *bves* variants or knockout lead to skeletal muscle atrophy. After an 8-week regular aerobic exercise intervention, we observed a significant increase in mitochondrial numerical density in the skeletal muscle of zebrafish, a rise in the percentage of normal-shaped mitochondria, and restoration of the expression levels of several genes related to oxygen concentration regulation. Additionally, regular aerobic exercise specifically enhanced the protein expression of mitochondrial complexes, indicating that it may improve mitochondrial oxidative respiratory function by enhancing the assembly efficiency of the terminal complexes in the electron transport chain. In summary, regular aerobic exercise can effectively ameliorate skeletal muscle atrophy and mitochondrial dysfunction caused by *bves* deficiency.

**Figure 10:**
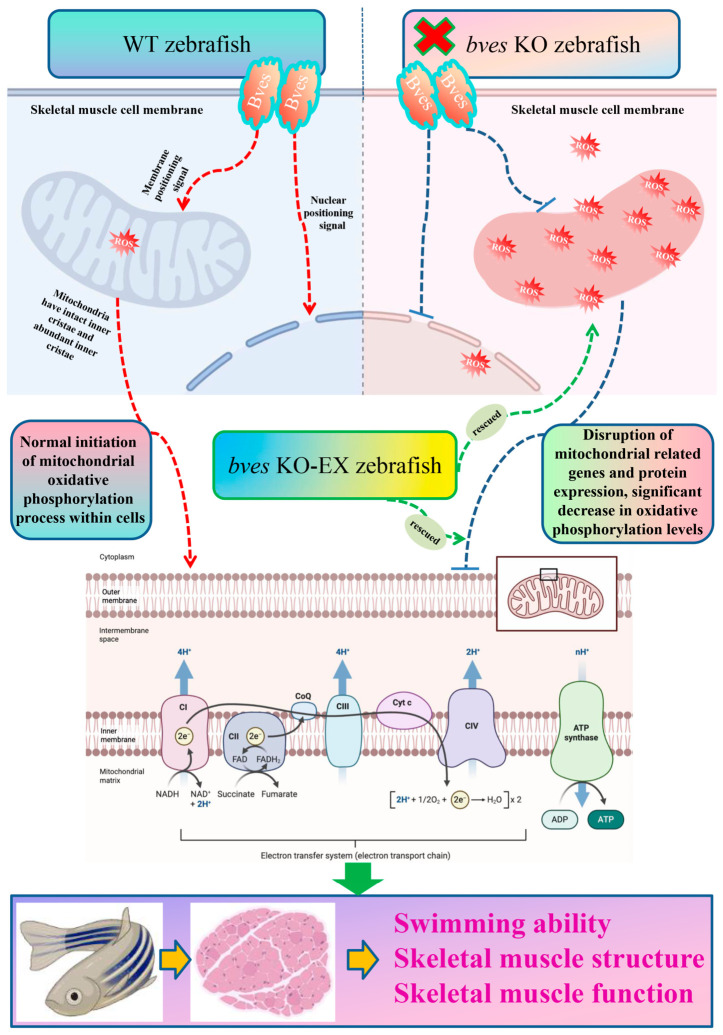
The mechanism through which Bves regulates mitochondrial function and skeletal muscle mass in response to skeletal muscle atrophy. The information depicted in this figure is sourced from Biorender (https://app.biorender.com/).

Human genetic disorders associated with Bves variants underscore the critical role of Bves in the maintenance of skeletal muscle mass and function. Variant or knockout of Bves are linked to LGMDR25, a primary cause of skeletal muscle atrophy. Under normal conditions, the Popeye domain-containing 1 (*popdc1*, *bves*) gene encodes a plasma membrane-localized cAMP-binding protein that is highly expressed in striated muscle. In animal models, Bves serves as an essential regulator of the structure and function of both cardiac and skeletal muscle. Research shows that the skeletal muscle of *popdc1*^S201F^ patients exhibits impaired membrane trafficking. Therefore, we hypothesize that in the WT group of zebrafish, Bves activates the expression of downstream mitochondria-related genes, promotes nuclear signaling, thereby maintaining the homeostasis of mitochondrial structure and function. In this context, the various complex proteins within the mitochondrial electron transport chain are normally expressed, and the oxidative phosphorylation process proceeds without disruption, with ROS production remaining within normal limits. Conversely, in *bves* KO zebrafish, the absence of *bves* leads to impaired downstream signaling, disruption of mitochondrial structure and function, significantly reduced levels of oxidative phosphorylation, excessive ROS production, and spillover into the cytoplasm and nucleus. This cascade of events results in skeletal muscle fiber atrophy and diminished swimming ability. Furthermore, an 8-week regular aerobic exercise intervention effectively rescues the expression levels of mitochondria-related genes and various complex proteins in the electron transport chain, leading to a partial restoration of mitochondria and skeletal muscle structure and function, alleviating symptoms of skeletal muscle atrophy and improving the swimming ability of zebrafish (Figure 10).

In summary, our results indicate that Bves is a crucial protein responsible for maintaining skeletal muscle mass and mitochondrial function. A deficiency in *bves* leads to severe mitochondrial dysfunction in skeletal muscle, damage to skeletal muscle fibers, and a decline in swimming ability in zebrafish. Regular aerobic exercise can effectively alleviate the pathological symptoms in skeletal muscle caused by the absence of *bves*. However, this study has some limitations: (1) Our research did not utilize zebrafish with skeletal muscle-specific *bves* knockout. (2) The direct impact of *bves* deficiency on the structure and function of mitochondria, a subcellular organelle in skeletal muscle, was not assessed. In *bves* KO zebrafish skeletal muscle, mitochondrial dysfunction and reduced oxidative respiratory capacity indicate impaired muscle metabolism. Whether the absence of *bves* further exacerbates skeletal muscle atrophy symptoms by affecting metabolism requires further exploration. Despite these limitations, we believe our study provides new insights into the mechanisms by which *bves* deficiency induces LGMDR25.

## 4. Materials and Methods

### 4.1. Animal Experiments

WT male and female zebrafish (AB strain) were obtained from the National Zebrafish Resource Center in Wuhan, China. The *bves* KO male and female zebrafish were generated in our laboratory using the CRISPR/Cas9 gene editing system. Following the crossbreeding of male and female zebrafish, the knockout effect was verified in the offspring, and 12-month-old male zebrafish were utilized for the experiments. All zebrafish were maintained in the Aisen Zebrafish Breeding System at the College of Life Sciences, Hunan Normal University. The rearing conditions included a water temperature of 28 ± 1 °C and a light/dark cycle of 14/10 h. Animal experiments were conducted in accordance with the “Guidelines for Proper Conduct of Animal Experiments” established by the Chinese Science Committee and received approval from the Ethics Committee of Hunan Normal University (Approval Number: 2025/886).

### 4.2. RNA Probe Synthesis and WISH

Whole-mount in situ hybridization (WISH) of zebrafish embryos [21,22] was conducted using digoxigenin-labeled antisense RNA probes. Total RNA was extracted from wild-type zebrafish tissues utilizing an RNA extraction kit (Fastagen, Shanghai, China, Cat#:220011), followed by the construction of a cDNA library using a reverse transcription kit (Takara, Kyoto, Japan, Cat#:RR036A-1). To create the probe, the exon sequence of the antisense probe for *bves* was amplified from cDNA via polymerase chain reaction (PCR) and subsequently transcribed into digoxigenin-labeled antisense RNA probes using an RNA transcription kit (Promega, Wi, USA, Cat#:P1440) along with Anti-Digoxigenin-AP Fab fragments (Roche, Basel, Switzerland, Cat#:11093274910). The primer sequences employed were as follows: *bves*-WISH-F: 5′-atccgcgctgccctccag-3′; *bves*-WISH-T7R:5′-CCCTAATACGACTCACTATAGGGgttgatccacctctcaggat-3′. Zebrafish embryos were subjected to fixation at certain developmental stages using a 4% paraformaldehyde solution (biosharp, Hefei, China, Cat#:BL539A). Following the fixation process, the embryos were preserved in 100% methanol (Foton, Wuhan, Hubei, Cat#:FP486B51602) to ensure the integrity of the samples for further analysis. To investigate the expression of the *bves* mRNA, WISH was performed. The results of this analysis were documented through images captured with a fluorescence microscope, specifically the Leica M205FA model, which is based in Wetzlar, Germany. The imaging process utilized Leica Application Kit software, version 3.2.0, to facilitate high-quality visual representations of the findings.

### 4.3. CRISPR/Cas9-Mediated bves Gene Knockout

The CRISPR/Cas9 gene editing system was employed to knock out the *bves* gene in zebrafish. To facilitate this process, polymerase chain reaction (PCR) was conducted using the P42250 plasmid as the template for synthesizing the single guide RNA (sgRNA). The reverse primer used in this reaction was sgRNA-R, a universal R-terminal primer specifically designed for the target site, which is detailed in Table S1. Additionally, two forward primers, sgRNA-primer-F1 and sgRNA-primer-F2, were also utilized; these primers contained both the T7 promoter and the target gene sequence necessary for effective sgRNA synthesis, as noted in Table S1. The synthesis of the sgRNA was carried out through in vitro transcription with the Riboprobe^®^ System-T7 Transcription Kit (Promega, Wi, USA, Cat#:P1440). The resulting sgRNAs were formulated at a final concentration of 20 ng/µL each. These sgRNAs were co-injected along with the Invitrogen TrueCut Cas9 v2 (Thermo Fisher Scientific, MA, USA, Cat#:A36499), which was used at a final concentration of 300 ng/µL, into one-cell-stage fertilized zebrafish eggs. The sgRNAs were specifically designed to exploit the capabilities of CRISPR/Cas9 technology to induce frameshift mutations within the coding sequence of the *bves* gene through the mechanism of non-homologous end joining [23,24,25]. This targeted approach resulted in the introduction of a frameshift mutation that created a premature stop codon, effectively halting protein translation. Following the injections, embryos that survived and showed signs of chimerism were mated with WT zebrafish. The subsequent offspring, which carried partial deletion mutant alleles of the *bves* gene, underwent sequencing to confirm the genetic modifications. The genotyping for the *bves* KO lines was effectively conducted using the designated forward and reverse primers, *bves*-KO-F and *bves*-KO-R, respectively, as illustrated in Table S1.

### 4.4. Behavioral Tests

The locomotor behavior of zebrafish embryos was monitored and analyzed using the high-throughput monitoring equipment of the ViewPoint ZebraLab zebrafish behavior analysis system [26] and its associated software. ZebraLab is an innovative research solution developed by ViewPoint, a company based in Lyon, France, specifically for zebrafish behavior research. This video tracking system enables the automated tracking and analysis of zebrafish movement, activity, and behavior, allowing for precise tracking and recording of the behavioral trajectories of 6 to 96 zebrafish embryos and larvae simultaneously. In this experiment, zebrafish larvae from different groups were placed into a 24-well cell culture plate containing embryo culture medium, with 12 wells designated for the WT control group larvae and the remaining 12 wells for the experimental group larvae, with one larva per well. The behavioral movements of zebrafish embryos were detected at 4, 6, 8, and 10 dpf. The total distance traveled by the larvae at different speeds, the total duration of movement at various speeds, and the number of movements within a 5 min period were recorded.

Behavioral analysis of adult zebrafish was conducted using LoliTrack (Tjele, Denmark) [27]. This device is equipped with a USB industrial color camera that monitors behavior by leveraging the distinct color contrast between the zebrafish and their surrounding environment. The measured parameters include speed, acceleration, travel distance, travel direction, effective and inactive time, as well as exhaustive swimming time. In summary, zebrafish are placed in a glass container filled with system water, and after a period of acclimatization, photographs are taken under a microscope in a quiet and undisturbed manner. Following the recording, the video is imported into LoliTrack software (version 4, Tjele, Denmark) for the analysis of the trajectory map, average speed, and total distance.

### 4.5. Critical Swimming Speed (Ucrit) and Exercise Training of Zebrafish

The critical swimming speed of zebrafish represents the maximum aerobic exercise capacity. It is generally believed that 40–60% Ucrit corresponds to moderate-intensity aerobic exercise, while approximately 80% Ucrit corresponds to high-intensity aerobic exercise. The Ucrit [28] of zebrafish was measured using the Loligo swim respirometer (Tjele, Denmark) [29], which is equipped with a DAQ-BT control unit and AutoRespTM software (version 2.2.2, Tjele, Denmark). The system consists of a 20 L water tank that contains a 170 mL enclosed swimming channel, supplying oxygenated recirculating water at a temperature of 28 ± 0.5 °C. Following a 24 h fasting period, zebrafish were placed in the Loligo swim respirometer for a 30 min acclimation period. Testing was conducted according to established procedures and parameters until the zebrafish reached exhaustion. The measurement data were subsequently exported, calculated, plotted, and analyzed. In this study, when devising a swimming exercise protocol for *bves* KO zebrafish, it was found that under the high-intensity aerobic exercise system, the water flow was turbulent, causing zebrafish to easily stick to the wall and avoid reverse swimming training. Therefore, a moderate-intensity (40–60% Ucrit) training mode was ultimately selected.

Twelve-month-old zebrafish with similar body size and weight were selected as experimental subjects. Under the same feeding conditions, they were divided into the WT group (*n* = 20), the *bves* KO group (*n* = 20), and the *bves* KO-EX group (*n* = 20). The exercise intervention protocol was optimized based on references [20,30]. Zebrafish in the *bves* KO-EX group underwent progressive training in swimming tunnels, with water flow velocity set at 40% of critical swimming speed (approximately 10 cm/s) during weeks 1–2, and gradually increased to 60% of the critical swimming speed (approximately 16 cm/s) during weeks 3–8. The training consisted of 3 consecutive days (1.5 h per day, including a 30 min adaptation period when flow velocity increased) followed by 1 rest day. During the training period, the WT and *bves* KO groups were housed in the same recirculating water system without electrical stimulation. Subsequently, the zebrafish were euthanized by rapid freezing in ice water. Skeletal muscle tissues were collected from the dorsal fin to the caudal fin region, immediately flash-frozen in liquid nitrogen, and stored at −80 °C for subsequent molecular biological analyses.

### 4.6. Histological Analysis

The skeletal muscle tissue of zebrafish was transversely sectioned into thin slices following a series of preparatory processes. H&E staining [31] and WGA staining [32] were performed to observe and quantify the size, Feret diameter, and area of skeletal muscle fibers. Masson staining was conducted to evaluate collagen deposition. ImageJ software (version 5.0) was utilized to calculate the area of muscle fibers. The calculation method was as follows: WGA-stained muscle fiber images were imported into ImageJ, and the image scale was set according to the scale information provided in the images. The images underwent preprocessing, and the threshold was adjusted to distinguish muscle fibers from the background. The “Analyze Particles” function, with appropriate parameters, was employed to automatically measure and label the area of muscle fibers, and the results were subsequently exported for analysis. The average muscle fiber area was determined from three randomly selected images per zebrafish, calculated based on 50 to 80 fibers per image.

### 4.7. Transmission Electron Microscope

Transmission electron microscopy [33,34] was employed to examine the skeletal muscle structure and its internal ultrastructure. Following the dissection process, zebrafish skeletal muscle tissues were subjected to a fixation procedure using an electron microscopy fixative for 24 h. This initial fixation step was crucial for preserving the structural integrity of the tissues. After fixation, the tissues were thoroughly washed with saline to eliminate any residual fixative, and subsequently immersed in a 1% osmium tetroxide solution for an additional 2 h. Osmium tetroxide acts as a secondary fixative that enhances contrast during electron microscopy imaging. Upon completion of the osmium tetroxide fixation, the tissues were washed repeatedly to ensure the removal of excess fixative. The samples were then subjected to a dehydration process before being embedded in epoxy resin, which provides a robust medium for further processing and fixation. The embedding procedure involved polymerizing the tissues in an oven maintained at 65 °C for 48 h, ensuring adequate permeation of the resin into the tissue. After polymerization, the resin-embedded tissues were sectioned into thin slices measuring 80 nanometers in thickness. The slices were subsequently stained with uranyl acetate and lead citrate, a procedure essential for enhancing electron density and contrast in the images obtained through transmission electron microscopy. In analyzing the resulting images, only mitochondria occupying more than half of the image area were counted as a single entity, while those occupying less than half were excluded from the analysis. To quantify mitochondrial area and length, ImageJ software (version 5.0) was utilized. The analytical process commenced by opening the representative mitochondrial images in ImageJ, where the image scale was calibrated using the straight-line tool. Subsequently, the straight-line tool was employed to measure mitochondrial length, and appropriate tools were selected to delineate the measurement area based on the unique shapes of the mitochondria. The final step involved exporting the measurement data for further analysis, ensuring precise quantification of mitochondrial parameters.

### 4.8. RNA-Seq-Based Transcriptome Analysis

Skeletal muscle tissues were dissected from various groups of zebrafish, with two skeletal muscle samples from each group placed in individual EP tube, constituting one biological replicate. Three tubes were randomly collected from each group and stored at −80 °C. Subsequent RNA sequencing was conducted at Majorbio (Shanghai, China), with data analysis was conducted on the Majorbio Cloud platform. Differentially expressed genes (DEGs) were analyzed using DESeq, applying screening criteria of |log2FC|> 2.0 and *p* < 0.05. The RNA-Seq results for the WT group, *bves* KO group, and *bves* KO-EX group can be found at the following website: https://www.ncbi.nlm.nih.gov/geo/ (GSE325211), accessed 20 May 2025. The volcano plot of DEGs was generated using three software packages: DESeq2 (version 1.57.1) [35], DEGseq (version 1.56.0) [36], and edgeR (version 4.0.1) [37]. Genes and transcripts exhibiting similar expression patterns may have analogous functions or be involved in the same metabolic processes or cellular pathways. Based on the expression levels of genes and transcripts across different samples, the distances between genes/transcripts or samples were calculated, followed by an iterative clustering approach. Gene Ontology (GO) enrichment analysis was performed using Goatools software (https://github.com/tanghaibao/GOatools, version 0.6.9, accessed 20 May 2025) [12]. When the adjusted *p*-value (FDR) was lower than 0.05, they were considered significantly enriched, sufficient to exhibit GO functions. The KEGG pathway enrichment analysis was conducted using the R language. The calculation principle was similar to that of GO function enrichment analysis, with *p* < 0.05 as the criterion.

### 4.9. ROS Detection

The measurement of ROS content by was conducted using DHE staining [30,38]. DHE penetrates cells freely and, in the presence of superoxide anions, undergoes dehydration to form ethidium bromide. This compound binds to RNA or DNA, resulting in the emission of red fluorescence. An elevated level of superoxide anions within cells leads to the generation of increased amounts of ethidium bromide, which correlates with a stronger red fluorescence signal. Briefly, the sections were outlined with an immunohistochemical pen, and a diluted DHE staining solution (Sigma, Darmstadt, Germany, Cat#:D7008) was applied to the marked areas. Nuclei were subsequently stained with DAPI staining solution. The positive areas were quantified using ImageJ software (Version 5.0). The quantification process involved converting the images to grayscale, followed by setting a threshold to differentiate positively stained areas from the background based on visual inspection and comparison with the original images. Regions of interest corresponding to the DHE-stained positive areas were automatically delineated. The parameters measured included area, average grayscale value, and threshold limits, which provided comprehensive insights into the distribution and intensity of ROS. The data were collected from five randomly selected images for each zebrafish.

### 4.10. Measurement of Mitochondrial Respiratory Function

Mitochondrial respiratory parameters in zebrafish skeletal muscle were measured using the Oxygraph-2K high-resolution respirometry system [39,40]. The muscle tissue was cryogenically ground and subsequently added to the sample chamber. Sequential additions of pyruvate, malate, glutamate, and ADP were performed to evaluate the activity of mitochondrial respiratory complex I (Cox I). Cytochrome C was incorporated to assess mitochondrial membrane integrity. Succinate was introduced to analyze the activities of mitochondrial complexes I and II (Cox I + II). The uncoupler CCCP was added to evaluate the capacity of the electron transport chain. Additionally, rotenone and antimycin A were injected to inhibit Cox I and Cox III, respectively.

### 4.11. Real-Time Quantitative PCR

To assess gene expression, RT-qPCR was utilized. Each group comprised three biological replicates (with each biological replicate representing skeletal muscle tissue dissected from an individual fish) and four technical replicates. Total RNA was extracted using the Novozyme Universal RNA Extraction Kit (Vazyme, Düsseldorf, Germany, Cat#:RC112-01). Subsequently, the total RNA was reverse transcribed into complementary DNA (cDNA) using the PrimeScript RT kit (Takara, Kyoto, Japan, Cat#:RR036A-1) as a template for RT-qPCR. The expression levels of target genes were normalized against the internal control *actb1* (also referred to as *β-actin*, which serves as a commonly used reference gene due to its relatively stable expression across various tissues and cell types. RT-qPCR was conducted using the QuantStudio 5 Real-Time PCR System (Thermo Fisher Scientific, Waltham, MA, USA) and QuantStudio Design along with Analysis Software (version 1.5.2). Detailed information regarding the primers for target genes is provided in Table S2. Relative gene expression values were calculated using the ΔΔCT method.

### 4.12. Western Blot Experiment

Total protein was extracted from skeletal muscle dissected from various groups of zebrafish. After weighing the tissues, they were homogenized under low-temperature conditions. A protein lysis buffer was added in proportion, followed by vortexing and centrifugation. The samples were then shaken for 10 min at 4 °C, sonicated, mixed with loading buffer through vortexing, boiled for 10 min, and centrifuged at 4 °C for an additional 10 min to collect the supernatant. Proteins were separated by SDS/PAGE and subsequently transferred onto PVDF membranes. The membranes were blocked with 5% milk at room temperature for 1 h and incubated with primary antibodies overnight at 4 °C. The following day, after washing the membranes three times with TBST solution, they were incubated with appropriate secondary antibodies for 1 h. After three additional washes, protein levels were detected using a gel imaging analyzer. Blots were normalized to Tubulin (marker band gradient is shown in Supplementary File S1—Original Protein Results). ImageJ software version 5.0 (NIH, Bethesda, MD, USA) was utilized for protein band analysis. Antibody information is provided in Table S3.

### 4.13. Statistical Analysis

Statistical analysis of the experimental data was performed using GraphPad Prism version 11.0.1. The data are presented as mean ± standard error of the mean (SEM). Comparisons between two groups were conducted using independent sample *t*-tests. Multiple comparisons among three groups were analyzed using one-way analysis of variance (ANOVA) followed by Tukey’s multiple comparison test. The significance threshold was set at *p* < 0.05.

## Data Availability

The original contributions presented in this study are included in the article/Supplementary Materials. Further inquiries can be directed to the corresponding authors.

## Supplementary Materials

The following supporting information can be downloaded at: https://www.mdpi.com/article/10.3390/ijms27125594/s1.
