# Supplementary material for: Regular Aerobic Exercise Can Effectively Ameliorate the Skeletal Muscle and Mitochondrial Function Impairments Caused by bves Deficiency in Zebrafish
Source: Int J Mol Sci. 2026 Jun 20;27(12):5594. doi: 10.3390/ijms27125594 (PMC13300094; doi:10.3390/ijms27125594)
Supplement: Supplementary file 1 [file ijms-27-05594-s001.zip › Supplementary File-Table S3.docx]

Table S3. Antibody sources and dilutions

| Antibodies | Source | Catalog | Dilution ratio |
| --- | --- | --- | --- |
| rabbit anti-Bves antibody | Proteintech, Wuhan, China | 12920-1-AP | 1:2000 |
| rabbit anti-Fbxo32 antibody | Huabio, Hangzhou, China | ET7109-25 | 1:2000 |
| rabbit anti-Ndufa4 antibody | Huabio, Hangzhou, China | ER64130 | 1:1000 |
| rabbit anti-Sdha antibody | Huabio, Hangzhou, China | ET1703-40 | 1:1000 |
| rabbit anti-Uqcrh antibody | Huabio, Hangzhou, China | HA720113 | 1:1000 |
| rabbit anti-Mt-co2 antibody | Abclonal, Wuhan, China | A3843 | 1:1000 |
| rabbit anti-Atp5a1 antibody | Proteintech, Wuhan, China | PK30006 | 1:1000 |
| rabbit anti-Tubulin antibody | MCE, New Jersey, United States | YA585 | 1:5000 |
