# Supplementary material for: Regular Aerobic Exercise Can Effectively Ameliorate the Skeletal Muscle and Mitochondrial Function Impairments Caused by bves Deficiency in Zebrafish
Source: Int J Mol Sci. 2026 Jun 20;27(12):5594. doi: 10.3390/ijms27125594 (PMC13300094; doi:10.3390/ijms27125594)
Supplement: Supplementary file 1 [file ijms-27-05594-s001.zip › Supplementary File S1-figures.docx]

**
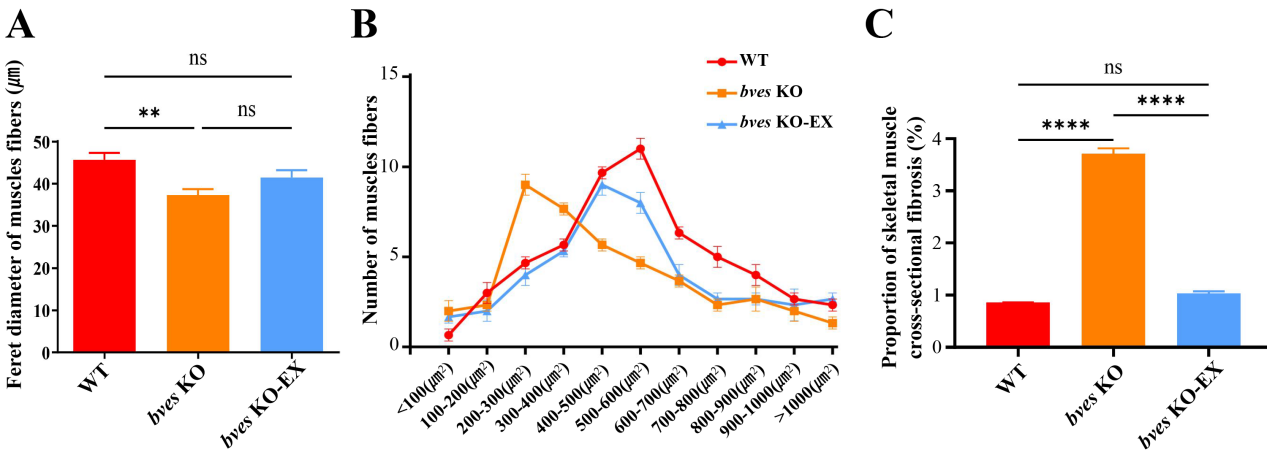
**

Figure S1. (A) Statistical results of the Feret diameter of skeletal muscle fibers (n = 20). (B) Distribution of individual muscle fiber cross-sectional area (CSA, n = 60). (C) Statistical results of the ratio of fibrosis area to skeletal muscle fiber cross-sectional area (n = 3). Statistical significance was determined using one-way analysis of variance (ANOVA), followed by Tukey's test for pairwise comparisons. Data are presented as mean ± standard error of the mean (SEM). ** *p* < 0.01, **** *p* < 0.0001, ns indicates no significant difference.


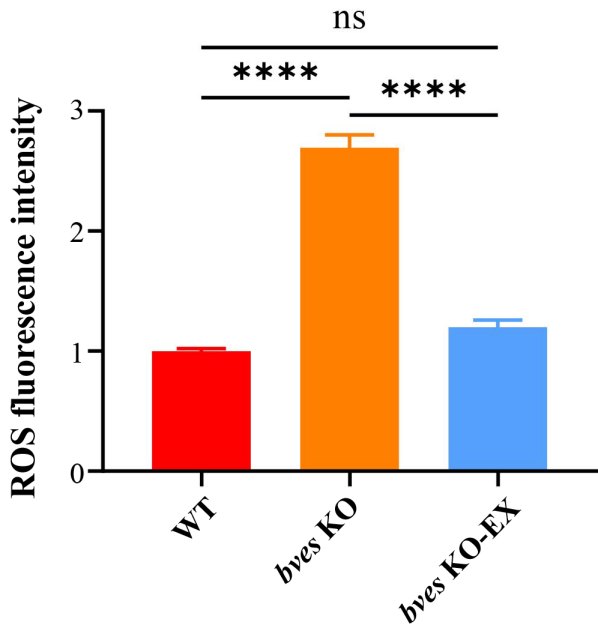


Figure S2. Quantitative results of dihydroethidium (DHE) staining fluorescence intensity (n = 3). Statistical significance was determined using one-way analysis of variance (ANOVA), followed by Tukey's test for pairwise comparisons. Data are presented as mean ± standard error of the mean (SEM). **** *p* < 0.0001, ns indicates no significant difference.


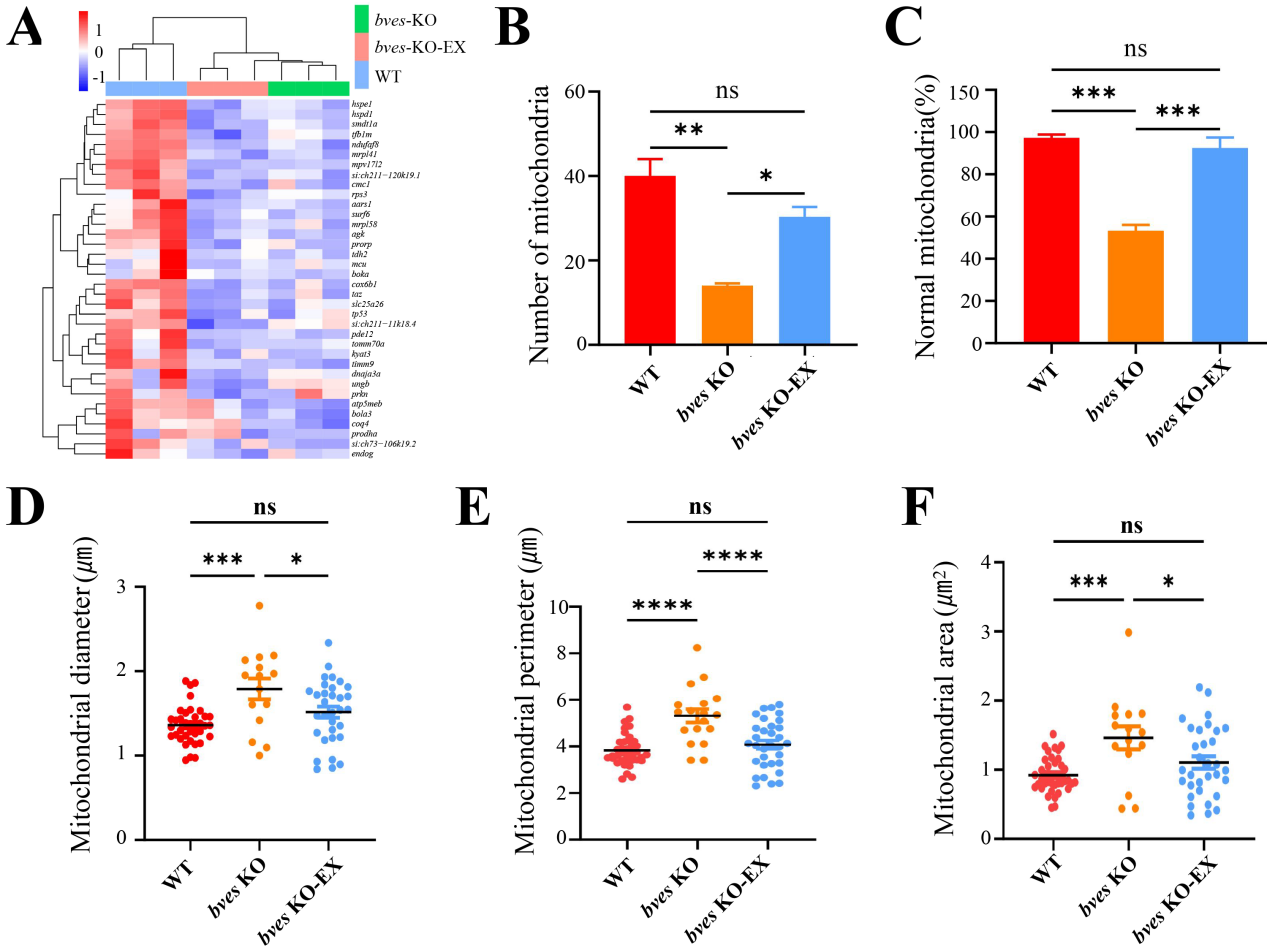


Figure S3. Loss of *bves* impairs mitochondrial structure and biogenesis in zebrafish skeletal muscle. (A) The heatmap displays differentially expressed genes (n = 3). (B) Statistical analysis of the number of mitochondria is presented (n = 3 fields of view). (C) The proportion of normal mitochondrial counts is shown (n = 3 fields of view). (D) Statistical results for the Feret diameter of mitochondria are summarized (n = 3 fields of view). (E) Statistical results concerning the perimeter of mitochondria are provided (n = 3 fields of view). (F) The statistical outcomes regarding mitochondrial area size are detailed (n = 3 fields of view). Statistical significance was determined using one-way analysis of variance (ANOVA), followed by Tukey's test for pairwise comparisons. Data are presented as mean ± standard error of the mean (SEM). * *p* < 0.05, ** *p* < 0.01, *** *p* < 0.001, **** *p* < 0.0001, ns indicates no significant difference.
