# Supplementary material for: Regular Aerobic Exercise Can Effectively Ameliorate the Skeletal Muscle and Mitochondrial Function Impairments Caused by bves Deficiency in Zebrafish
Source: Int J Mol Sci. 2026 Jun 20;27(12):5594. doi: 10.3390/ijms27125594 (PMC13300094; doi:10.3390/ijms27125594)

Fogure 9A: Fbxo32 (41KDa)

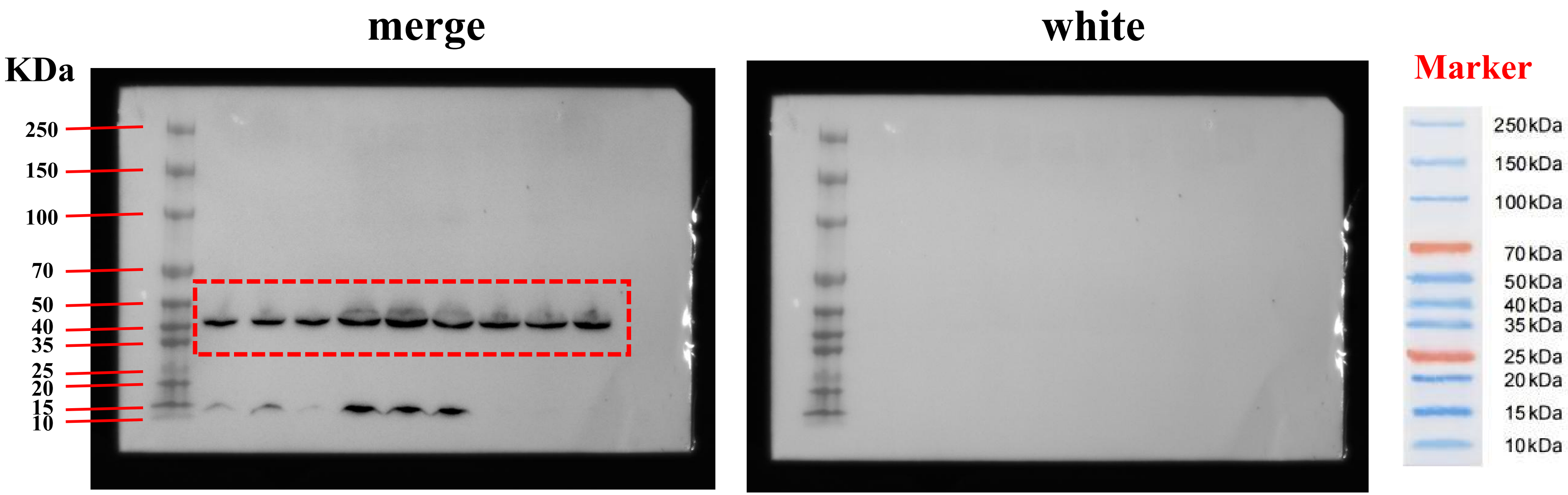

Fogure 9A:Ndufa4 (9KDa)

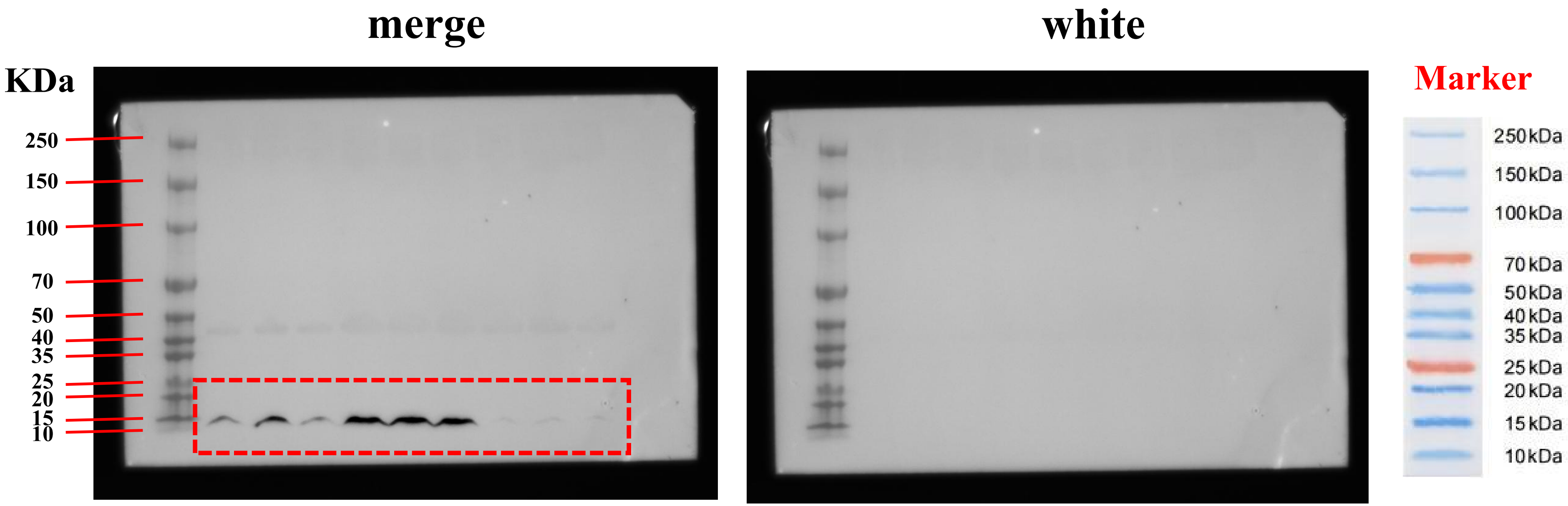

Fogure 9A:Sdha (73KDa)

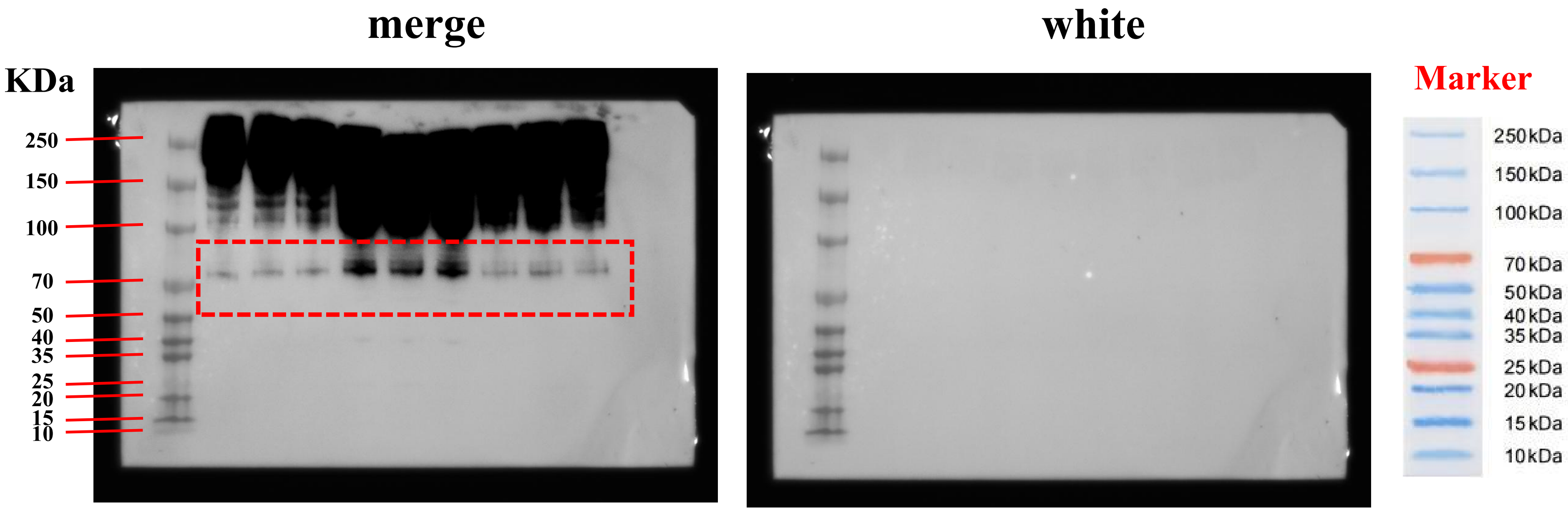

Fogure 9A:Uqcrh (14KDa)

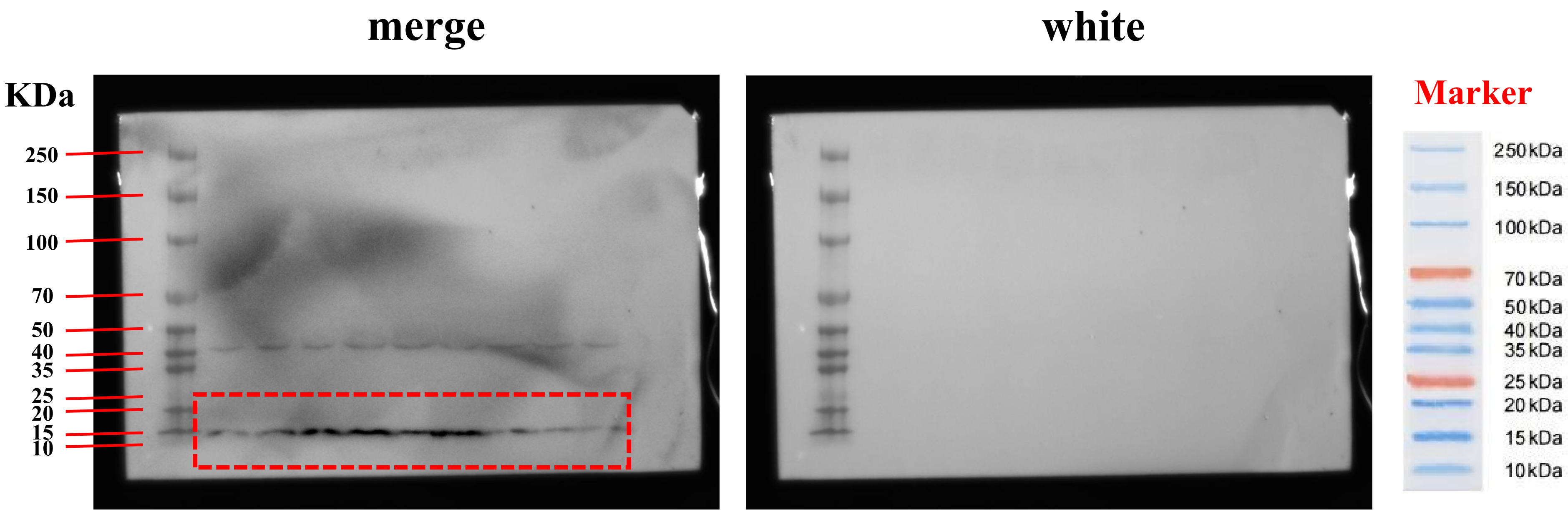

Fogure 9A:Mtco2 (23KDa)

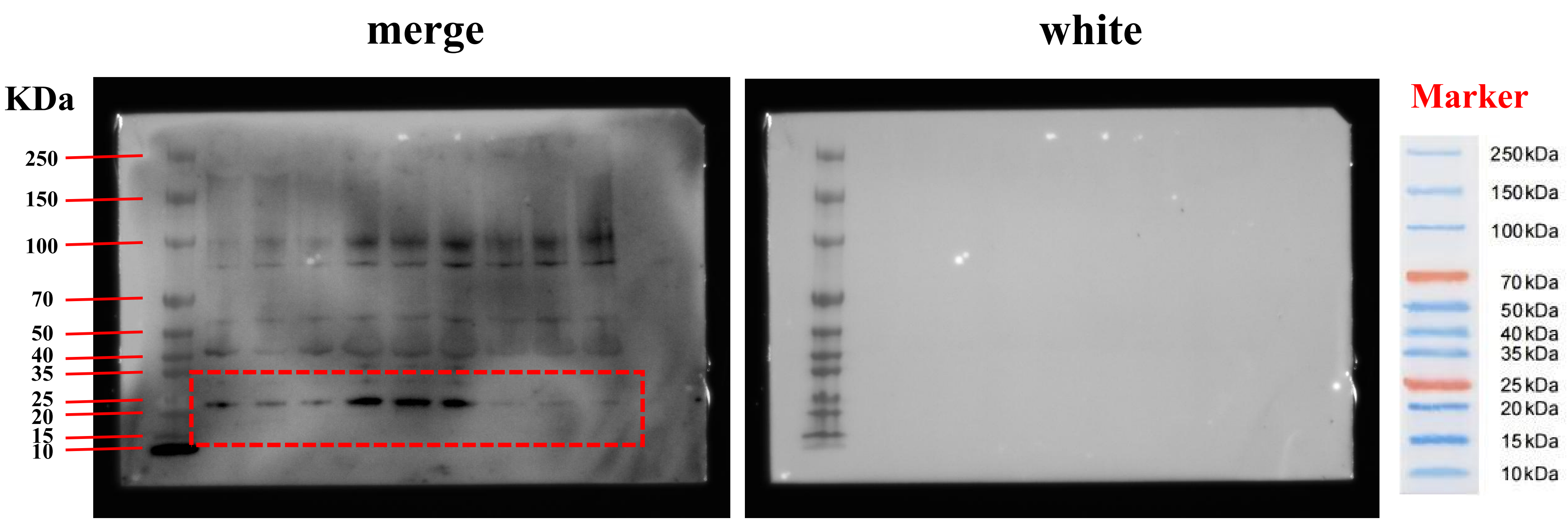

Fogure 9A:Atp5a1 (57KDa)

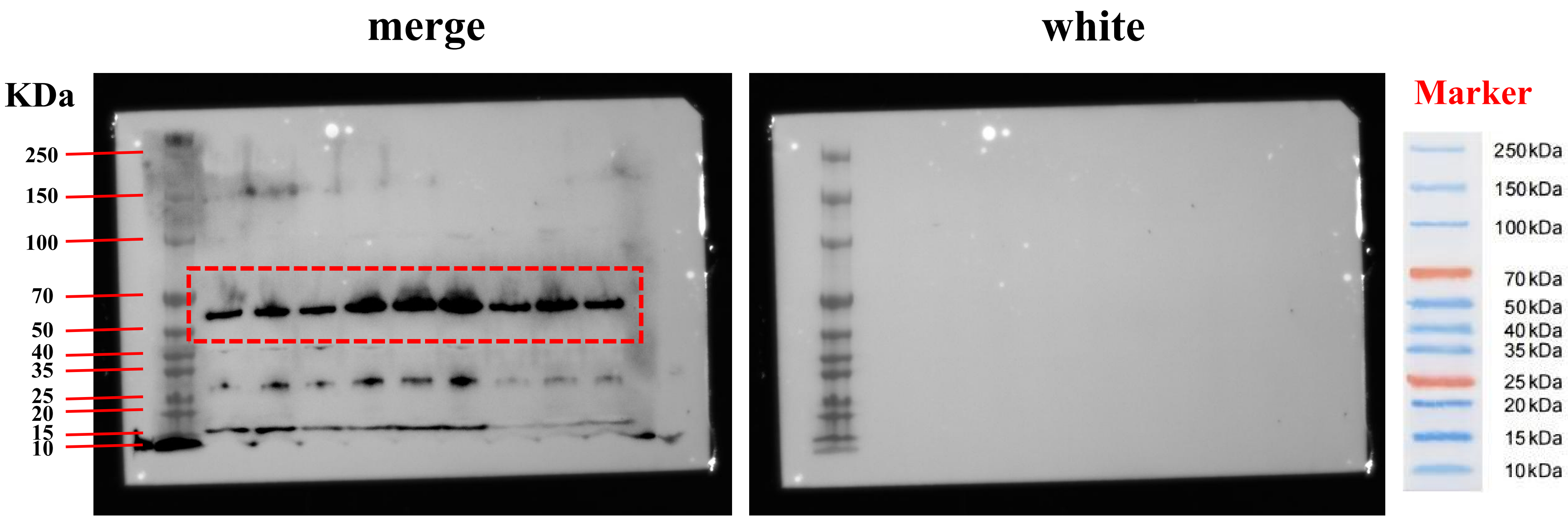

Fogure 9A:Tubulin(53KDa)

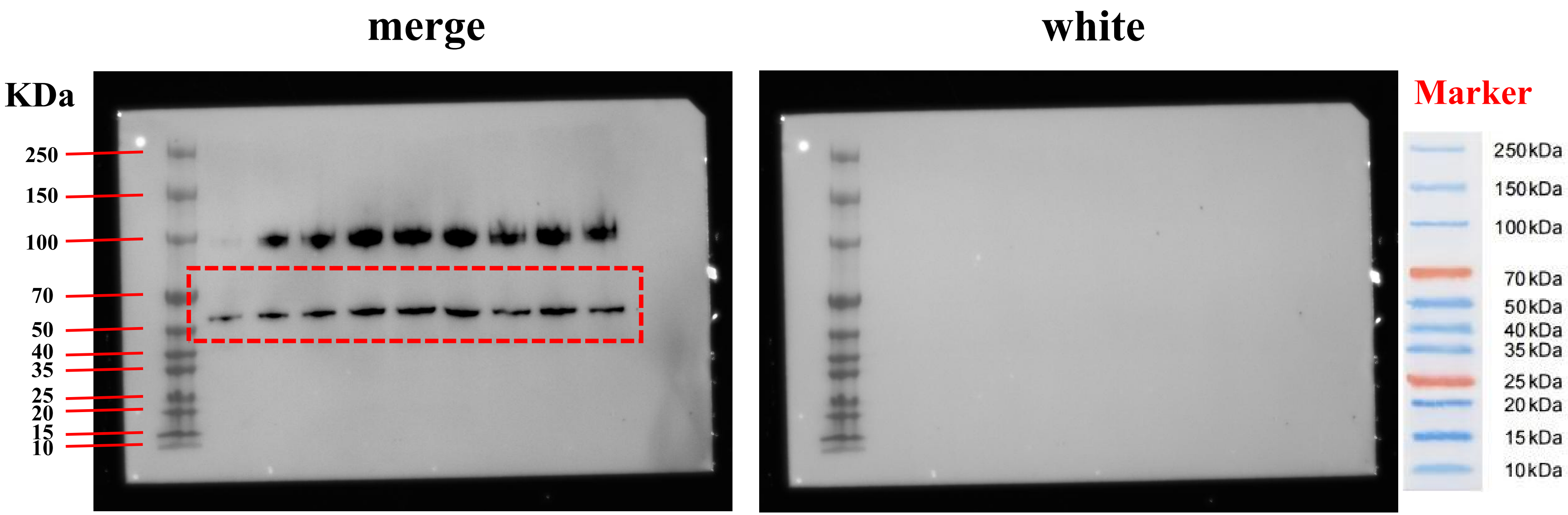

Supplement: Supplementary file 1 [file ijms-27-05594-s001.zip › Supplementary File S1-Original protein results.pdf]
