# Supplementary material for: Regular Aerobic Exercise Can Effectively Ameliorate the Skeletal Muscle and Mitochondrial Function Impairments Caused by bves Deficiency in Zebrafish
Source: Int J Mol Sci. 2026 Jun 20;27(12):5594. doi: 10.3390/ijms27125594 (PMC13300094; doi:10.3390/ijms27125594)
Supplement: Supplementary file 1 [file ijms-27-05594-s001.zip › Supplementary File-Table S1.docx]

Table S1. Primers used for *bves* gene knockout and their sequences

| Primer | Sequences (5′–3′) |
| --- | --- |
| sgRNA-primer-F1 | tgTAATACGACTCACTATAGGacccagcccgagactcaggtGTTTTAGAGCTAGAAATAGC |
| sgRNA-primer-F2 | tgTAATACGACTCACTATAGGtctattggtgctctacgggaGTTTTAGAGCTAGAAATAGC |
| sgRNA-R | aaGCACCGACTCGGTGCCACT |
| *bves*-KO-F | ACGCCACACTATGCCAGGAT |
| *bves*-KO-R | ATTCTGACTTCAAATTTATATATACCC |
