# Supplementary material for: Regular Aerobic Exercise Can Effectively Ameliorate the Skeletal Muscle and Mitochondrial Function Impairments Caused by bves Deficiency in Zebrafish
Source: Int J Mol Sci. 2026 Jun 20;27(12):5594. doi: 10.3390/ijms27125594 (PMC13300094; doi:10.3390/ijms27125594)
Supplement: Supplementary file 1 [file ijms-27-05594-s001.zip › Supplementary File-Table S2.docx]

Table S2. List of primers used for RT-qPCR

| Gene | Accession number | Forward primer (5′–3′) | Reverse primer (5′–3′) |
| --- | --- | --- | --- |
| *actb1* | [NM_131031.2](https://www.ncbi.nlm.nih.gov/nuccore/NM_131031.2) | TCCAGCCTTCCTTCCTGGGTAT | GTGGAAGGAGCAAGAGAGGTG |
| *bves* | [NM_001001847.2](https://www.ncbi.nlm.nih.gov/nuccore/NM_001001847.2) | AGCGCCTCAGCATTCTTCTC | AAAGCGTTCACCCCTGTTCA |
| *bcl6b* | [XM_001332966.9](https://www.ncbi.nlm.nih.gov/nuccore/XM_001332966.9) | AGCCAGTCTTCAGAACAGCC | TAGCGACGCAAAGCTCTCAT |
| *foxo4* | [XM_009291168.4](https://www.ncbi.nlm.nih.gov/nuccore/XM_009291168.4) | TGATGACCCAGATGGACCCT | CCACCGTGCTTGTGTCAATG |
| *mapk6* | [XM_073929323.1](https://www.ncbi.nlm.nih.gov/nuccore/XM_073929323.1) | AACGTGCTTCATCGTGACCT | TGGGGGATAGAAGCAGACGA |
| *pik3r3a* | [NM_001291329.1](https://www.ncbi.nlm.nih.gov/nuccore/NM_001291329.1) | GGGCGGACATATCAAGGGAG | TTTGTTCGTGCCGTTGTTCC |
| *sgk2b* | [NM_001076564.1](https://www.ncbi.nlm.nih.gov/nuccore/NM_001076564.1) | AAGAGTCCCGTCACTTACGC | GTCGCAAGAAATCCATCTGGTG |
| *atp5meb* | [NM_001098481.1](https://www.ncbi.nlm.nih.gov/nuccore/NM_001098481.1) | GCGTTGCTCGTTGGCATTAT | GCTAGCTGTTTAGCGATGCG |
| *ddit3* | [NM_001082825.1](https://www.ncbi.nlm.nih.gov/nuccore/NM_001082825.1) | TTCCCGACACATCATCCTGC | TTCATTCTCCTGTTCGCGCT |
| *epas1a* | [XM_690170.11](https://www.ncbi.nlm.nih.gov/nuccore/XM_690170.11) | TGTTCCACCACCACACCAAA | AGCGACCACCTGTTTTGGAA |
| *hif1al* | [NM_200405.1](https://www.ncbi.nlm.nih.gov/nuccore/NM_200405.1) | GCTTCTGCTCAGCCACACAT | CGGGTCCCTGTCGGTTAAAA |
| *hmox1a* | [NM_001127516.1](https://www.ncbi.nlm.nih.gov/nuccore/NM_001127516.1) | GCAGGACTTGGAGCACTTCT | GGGACTGCTCTTGCCAATCT |
| *igfbp1a* | [NM_173283.4](https://www.ncbi.nlm.nih.gov/nuccore/NM_173283.4) | CGCGATACGCAAGAAACTGG | CAACACTTCCCCCTCTGACC |
| *irs1* | [XM_682610.11](https://www.ncbi.nlm.nih.gov/nuccore/XM_682610.11) | TGACTGCCTCTTTCCACGTC | CTTCGAAAGTCACAGGGGCT |
| *irs2a* | [XM_021466764.3](https://www.ncbi.nlm.nih.gov/nuccore/XM_021466764.3) | CAGTCAGCCCCACTAACAGG | GGTCTCAGAGCTATGCCGTC |
| *loxl2a* | [NM_001099244.1](https://www.ncbi.nlm.nih.gov/nuccore/NM_001099244.1) | GTCTCCTGCTCTGATACGGC | TCATTCTTTCGGGCAGTGCT |
| *nrp1a* | [NM_001040326.1](https://www.ncbi.nlm.nih.gov/nuccore/NM_001040326.1) | GACGTGAGCTCCAATGGTGA | CATCCGTAGACCTCGAACCG |
