# Supplementary figures and images for: Regular Aerobic Exercise Can Effectively Ameliorate the Skeletal Muscle and Mitochondrial Function Impairments Caused by bves Deficiency in Zebrafish
Source: Int J Mol Sci. 2026 Jun 20;27(12):5594. doi: 10.3390/ijms27125594 (PMC13300094; doi:10.3390/ijms27125594)

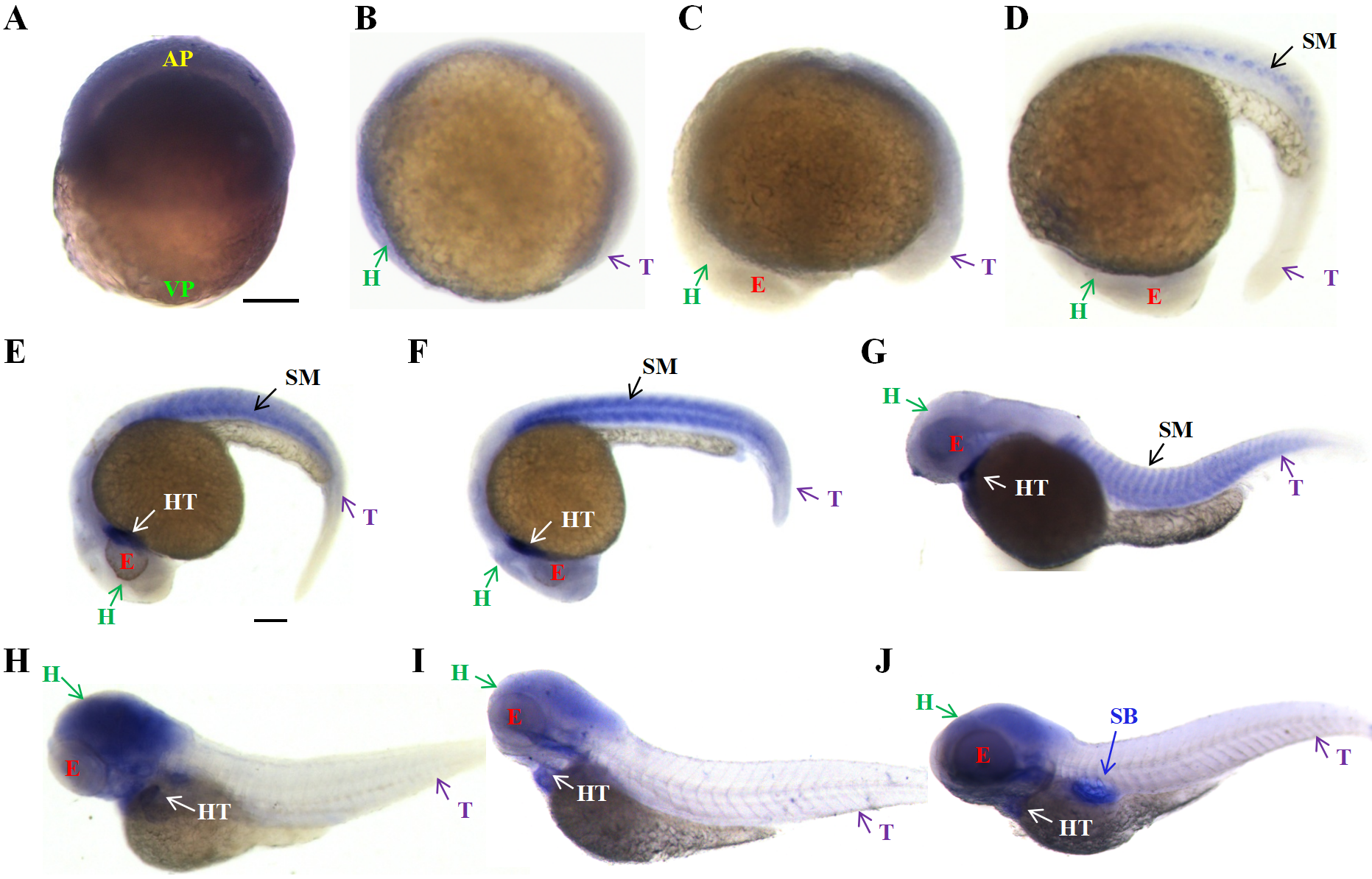

Supplement: Supplementary file 1 [file ijms-27-05594-s001.zip › Figure 1.tif]

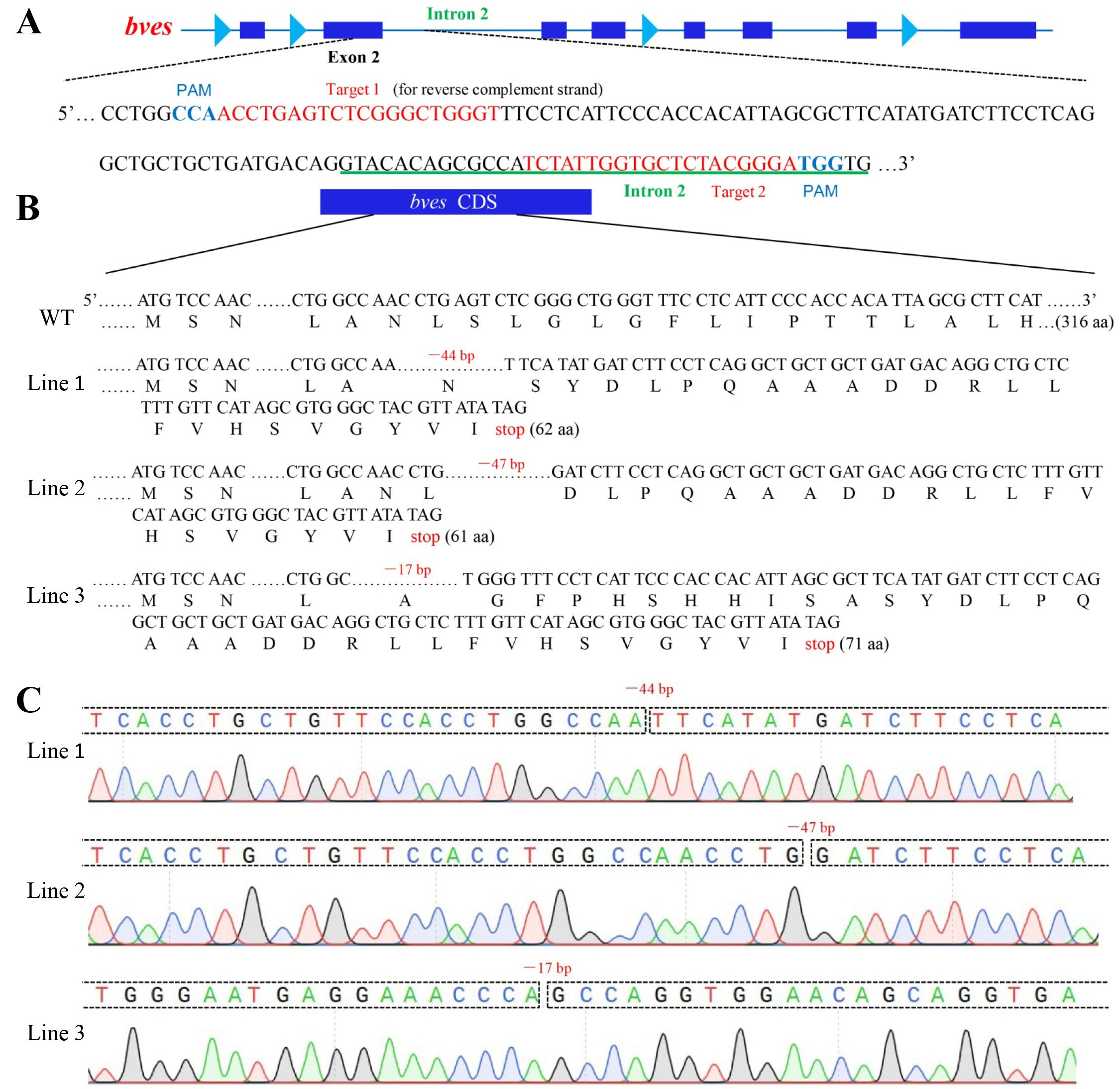

Supplement: Supplementary file 1 [file ijms-27-05594-s001.zip › Figure 2.jpg]

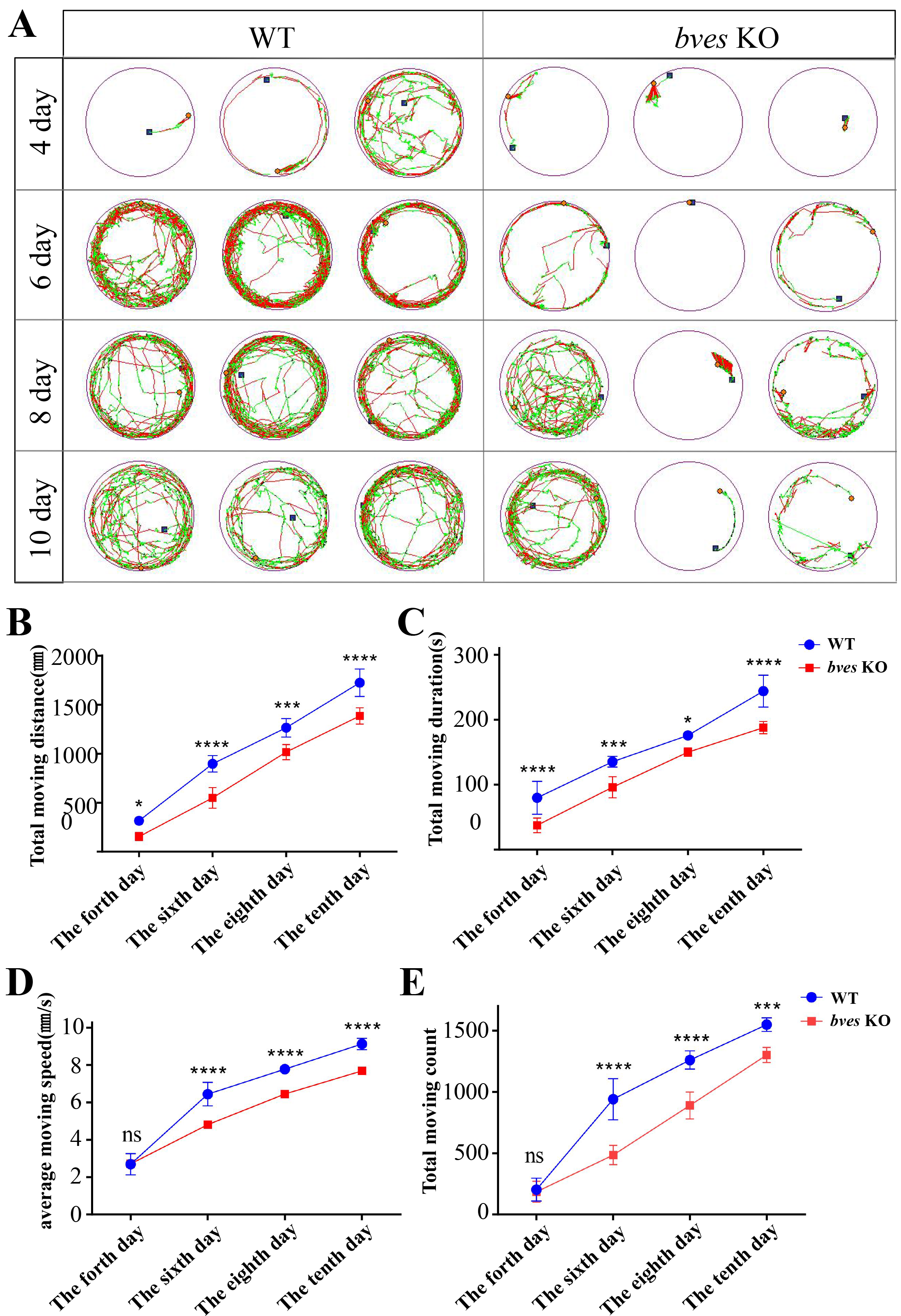

Supplement: Supplementary file 1 [file ijms-27-05594-s001.zip › Figure 3.tif]

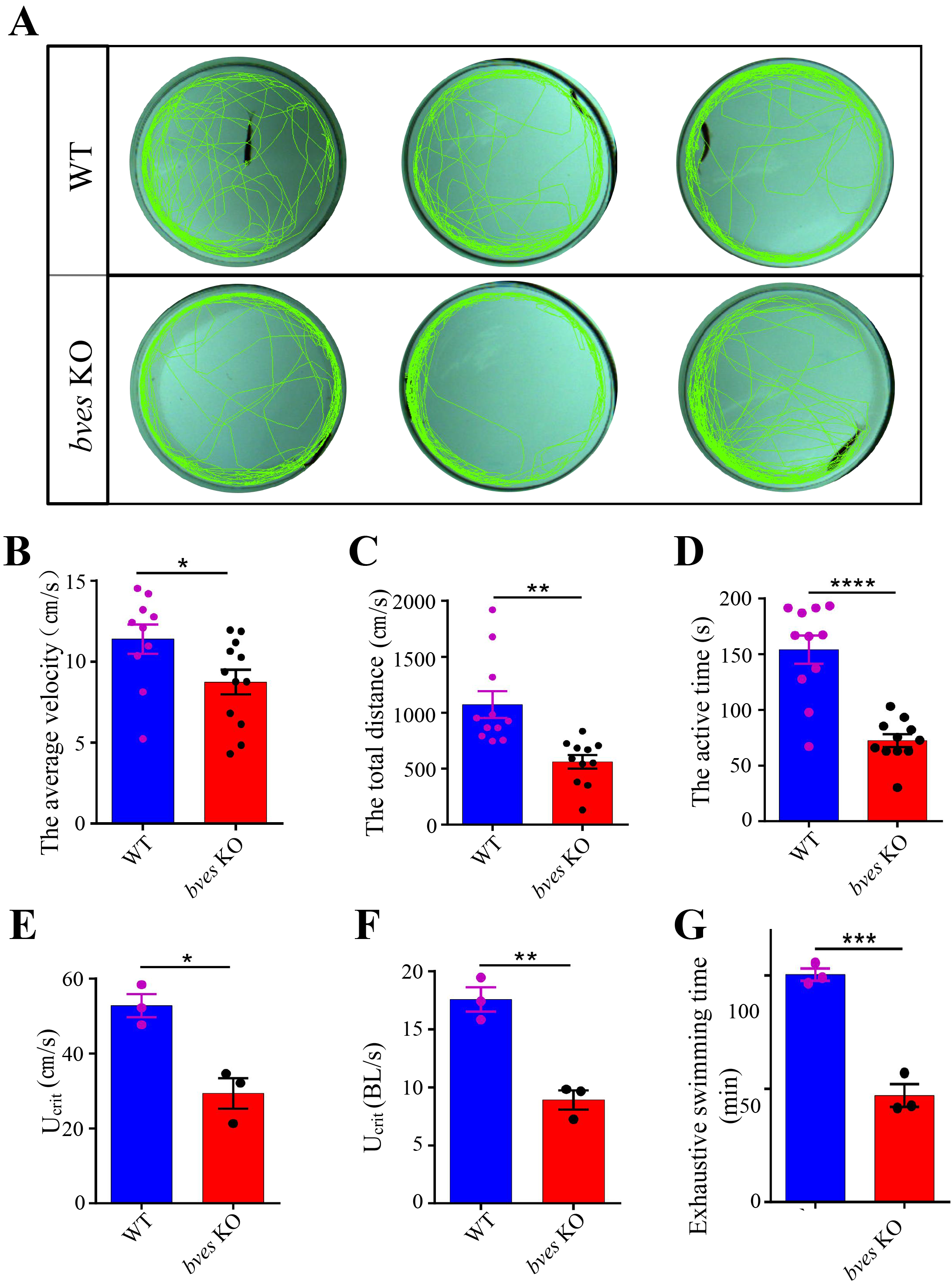

Supplement: Supplementary file 1 [file ijms-27-05594-s001.zip › Figure 4.tif]

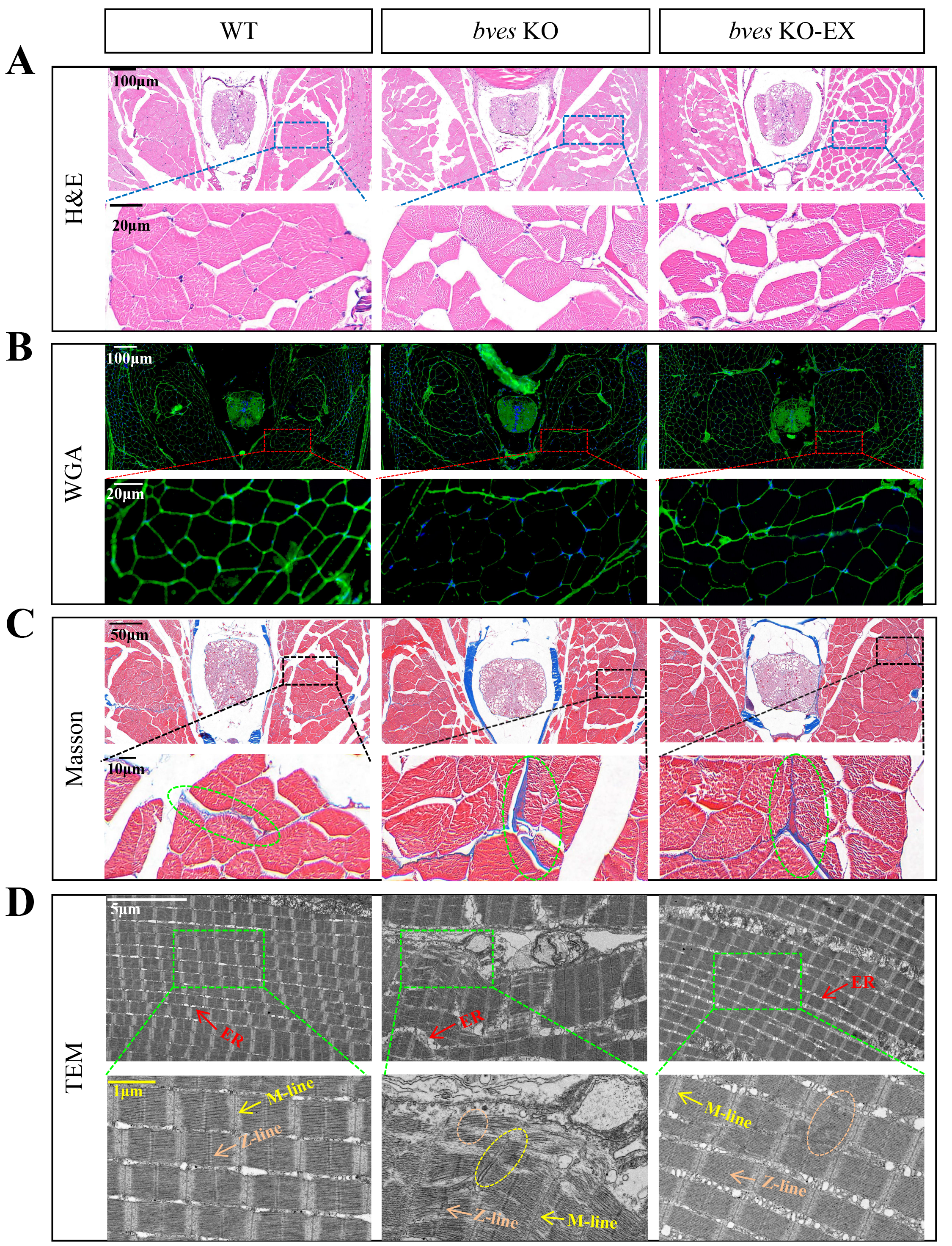

Supplement: Supplementary file 1 [file ijms-27-05594-s001.zip › Figure 5.tif]

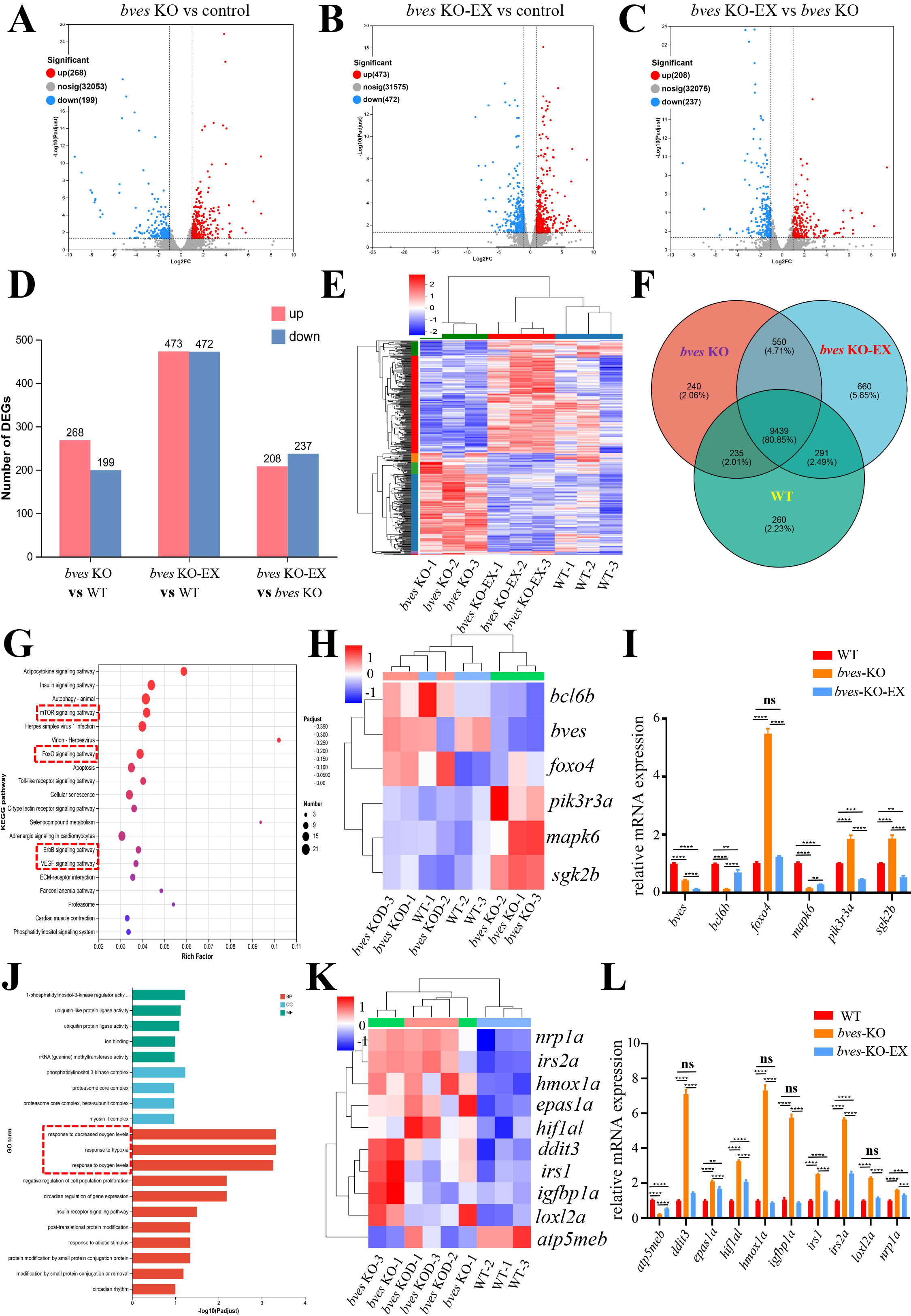

Supplement: Supplementary file 1 [file ijms-27-05594-s001.zip › Figure 6.tif]

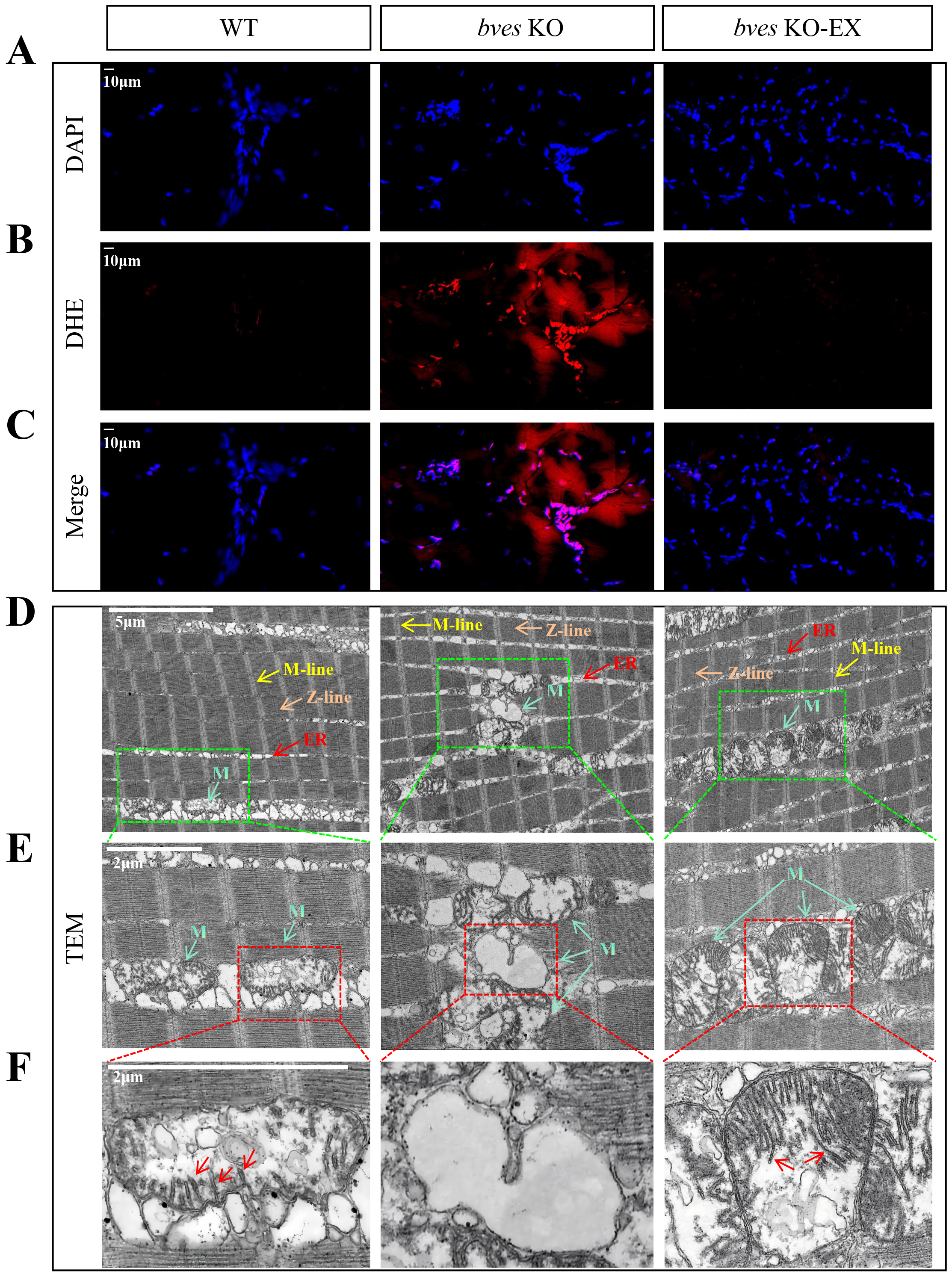

Supplement: Supplementary file 1 [file ijms-27-05594-s001.zip › Figure 7.tif]

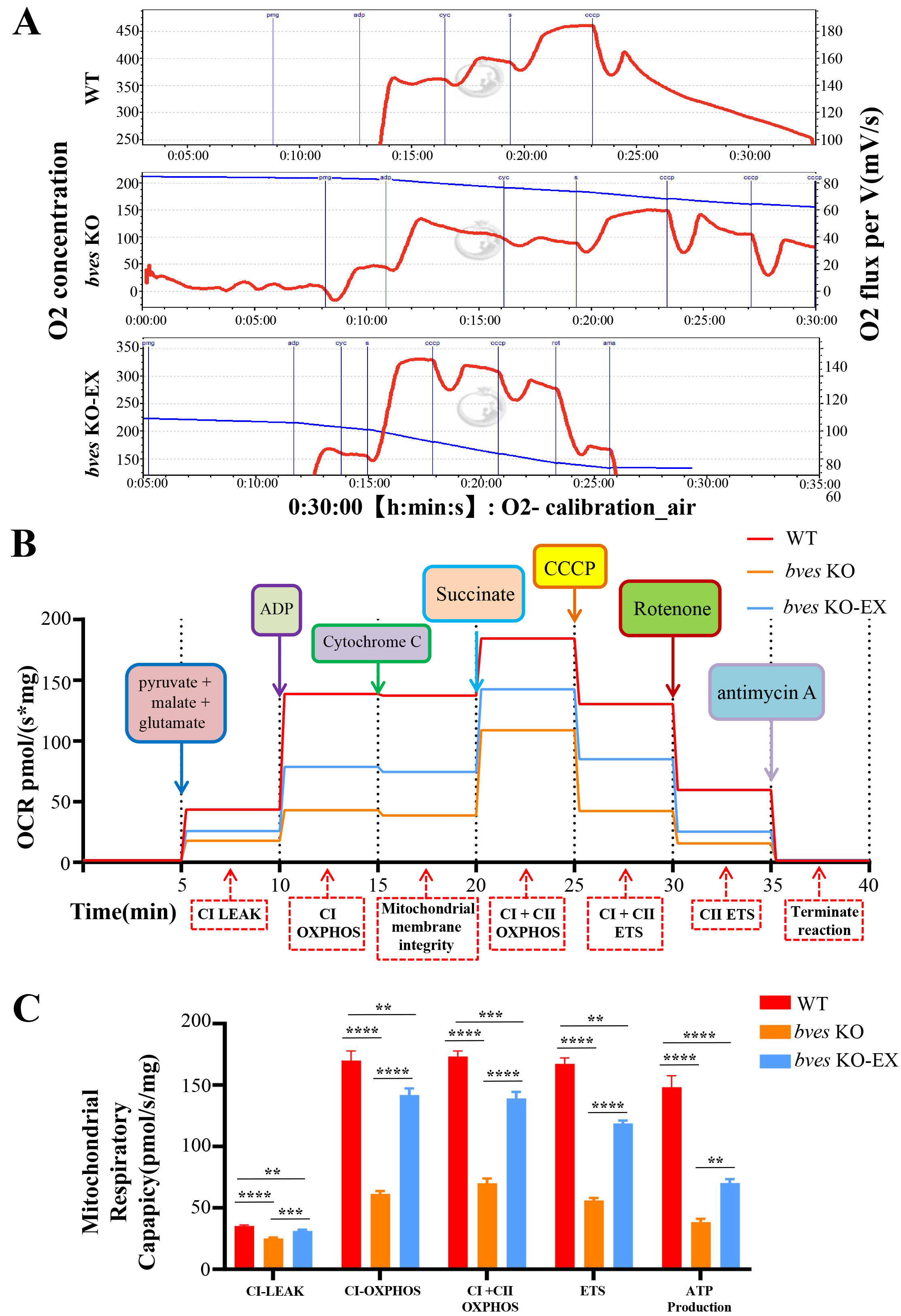

Supplement: Supplementary file 1 [file ijms-27-05594-s001.zip › Figure 8.tif]

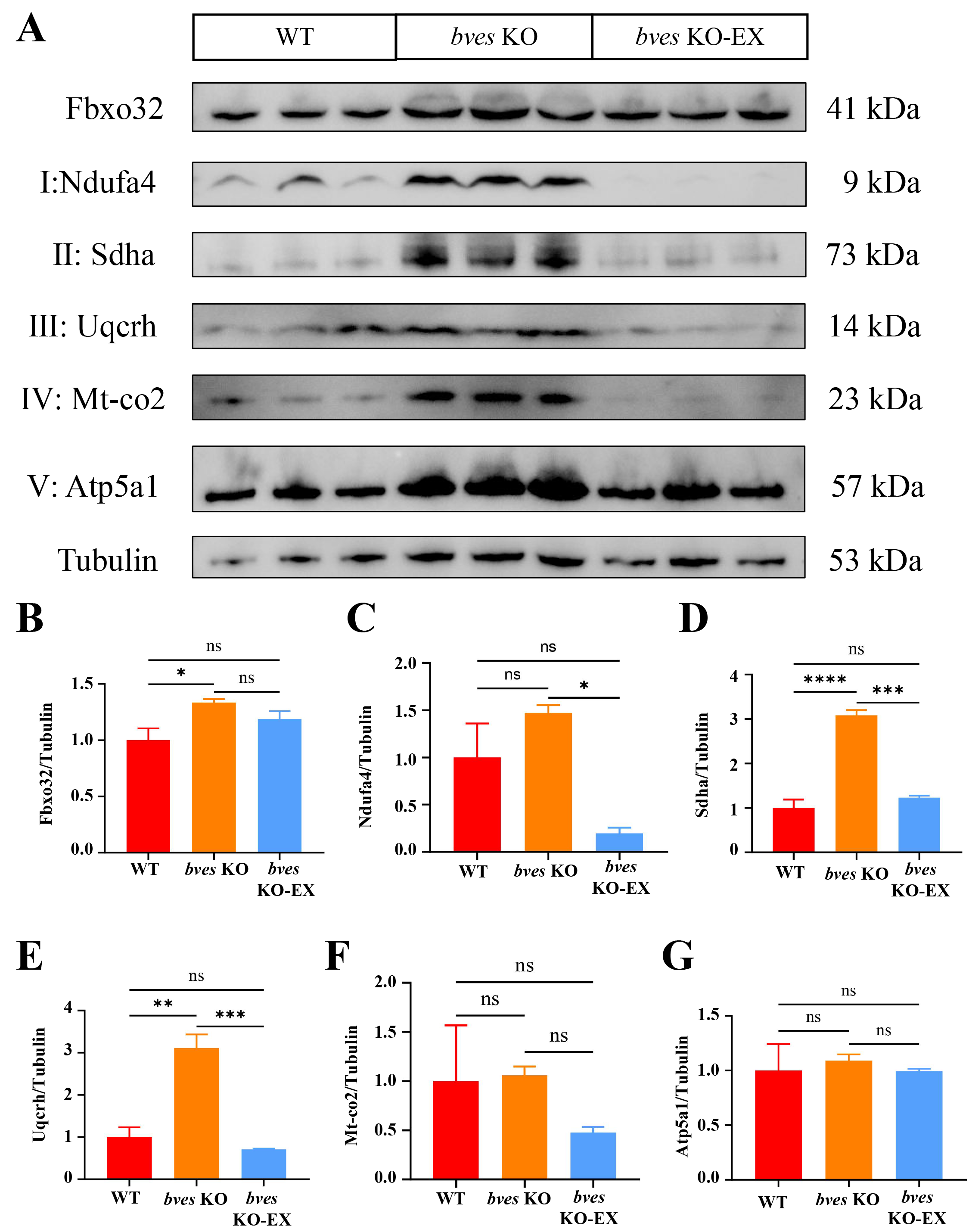

Supplement: Supplementary file 1 [file ijms-27-05594-s001.zip › Figure 9.tif]

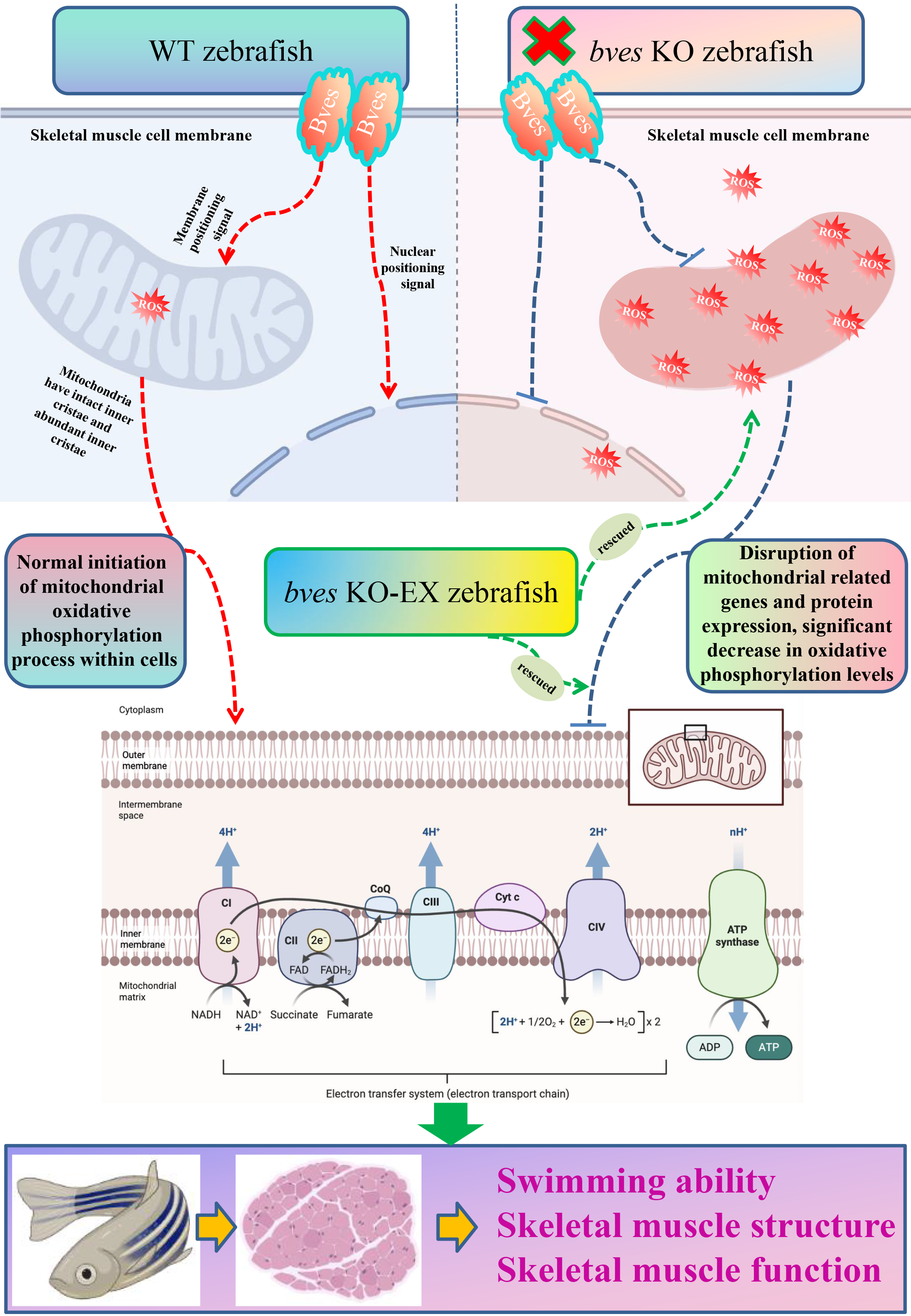

Supplement: Supplementary file 1 [file ijms-27-05594-s001.zip › Figure 10.tif]

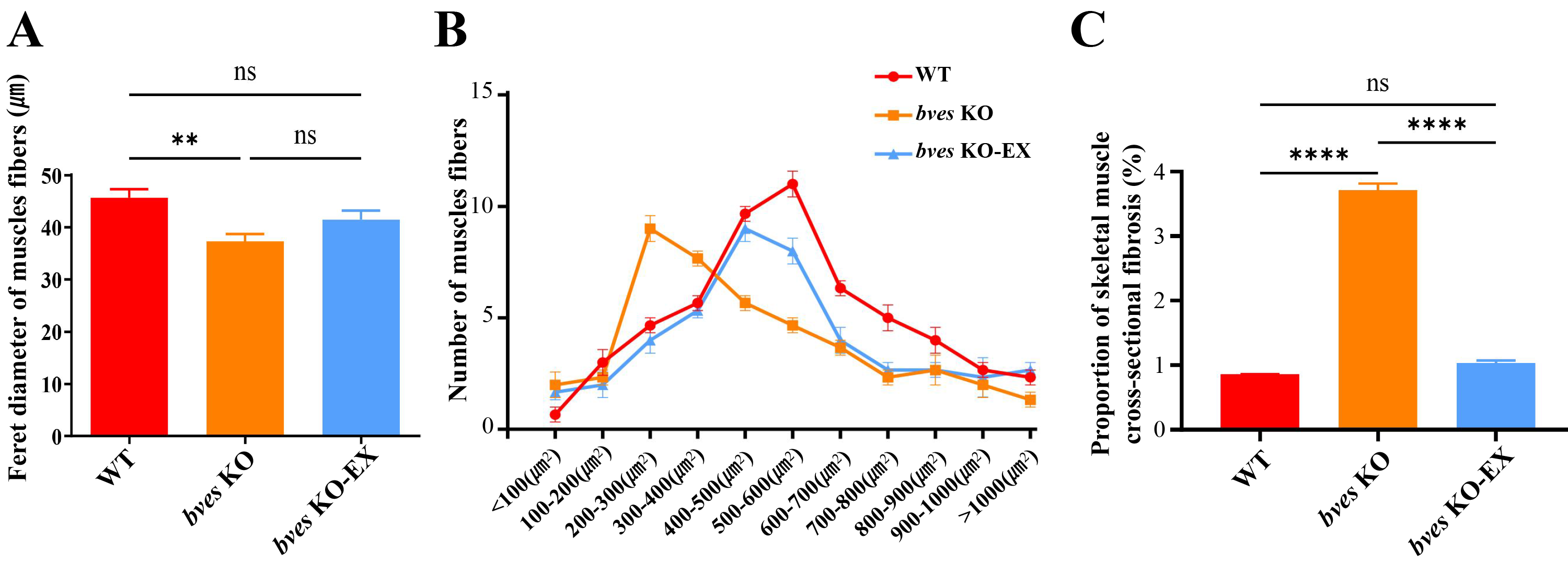

Supplement: Supplementary file 1 [file ijms-27-05594-s001.zip › Figure S1.tif]

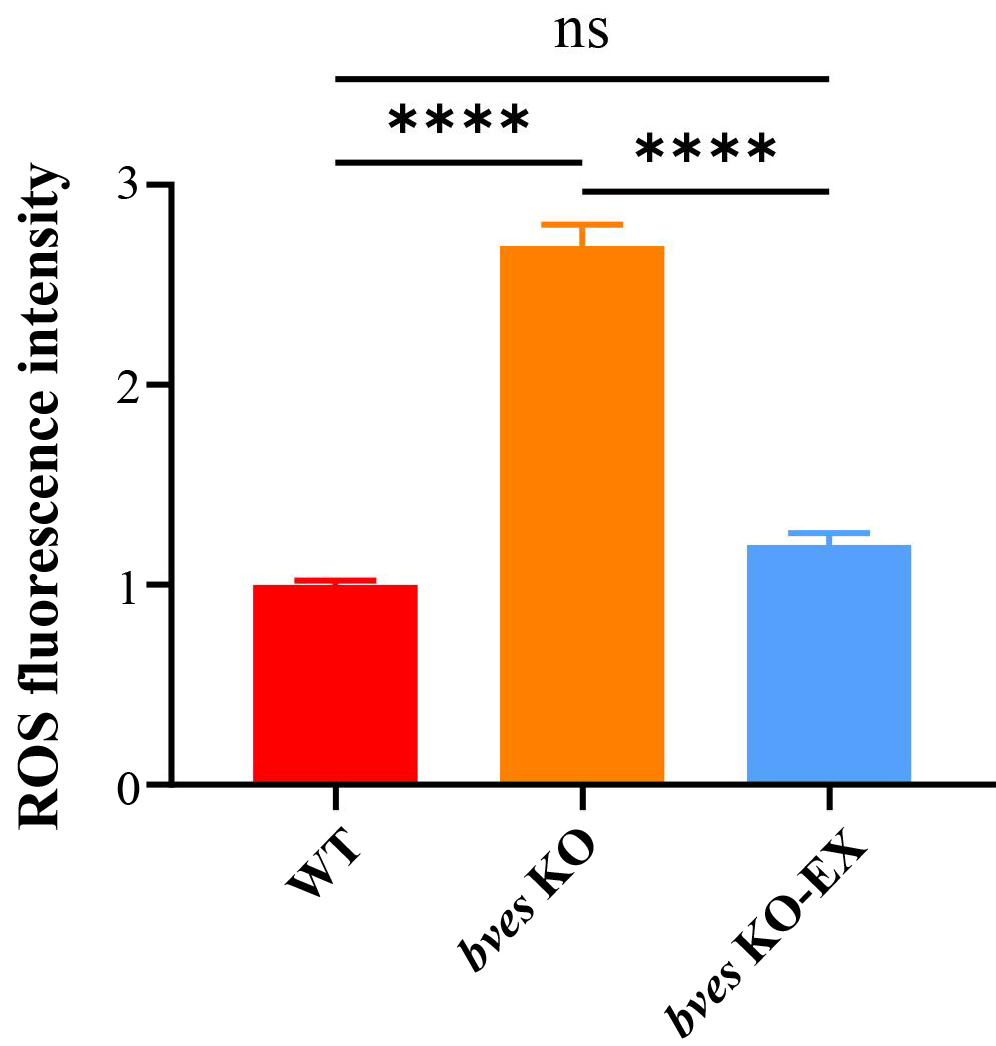

Supplement: Supplementary file 1 [file ijms-27-05594-s001.zip › Figure S2.tif]

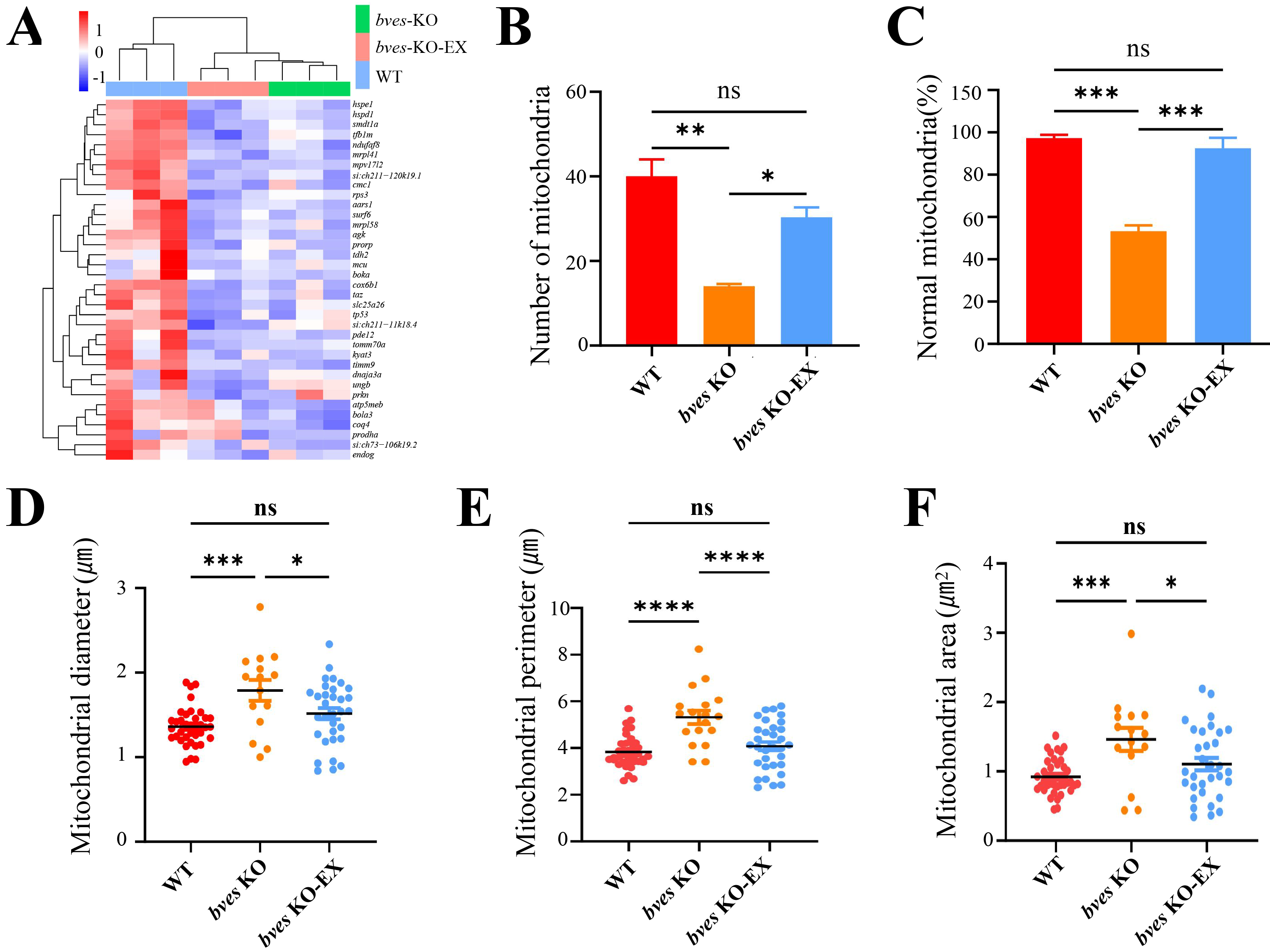

Supplement: Supplementary file 1 [file ijms-27-05594-s001.zip › Figure S3.tif]
